# A generative framework for estimating brain energy landscapes from continuous neuroimaging observations

**DOI:** 10.1162/IMAG.a.1415

**Published:** 2026-10-27

**Authors:** Seok-Oh Jeong, Euisun Kim, Jiyoung Kang, Jinseok Eo, Dongmyeong Lee, Hae-Jeong Park

**Affiliations:** Department of Statistics, Hankuk University of Foreign Studies, Yong-In, Republic of Korea; Graduate School of Medical Science, Brain Korea 21 Project, Department of Nuclear Medicine, Department of Psychiatry, Yonsei University College of Medicine, Seoul, Republic of Korea; Center for Systems and Translational Brain Science, Institute of Human Complexity and Systems Science, Yonsei University, Seoul, Republic of Korea; Department of Scientific Computing, Pukyong National University, Busan, Republic of Korea; Department of Cognitive Science, Yonsei University, Seoul, Republic of Korea; Brain Research Institute, Epilepsy Research Institute, Yonsei University College of Medicine, Seoul, Republic of Korea

**Keywords:** pairwise maximum entropy model, nonlinear state dynamics, latent binary states, correlated noise, EEG/MEG source, energy landscape

## Abstract

Pairwise maximum entropy models (pMEMs) are gaining increasing attention as a principled way to study nonlinear brain-state transitions. By representing each brain region as a binary active/inactive state and estimating the least-biased probability distribution over all such network configurations, pMEMs reconstruct an energy landscape in which local minima correspond to stable or metastable brain states and transitions reflect movement across attractor basins. However, neuroimaging recordings are continuous and spatially mixed, forcing conventional pMEM analyses to demean and hard-threshold each channel. This ad hoc binarization discards amplitude information and can conflate spatially correlated noise or source mixing with genuine neural coupling. We introduce a hierarchical generative pMEM that links latent binary network states to continuous observations. Node-specific baseline and gain parameters model amplitude modulation, compound-symmetry noise captures shared residual covariance, and, for electroencephalography/magnetoencephalography (EEG/MEG), the electromagnetic leadfield is included as a generative forward model rather than a preprocessing inverse. Model parameters and latent states are jointly estimated using exact posterior enumeration for the small networks analyzed here and a mean-field extension for larger networks. Controlled simulations demonstrated two complementary advantages of the proposed framework. Residual-covariance modeling improved recovery of pMEM fields, couplings, and latent states by accounting for shared fluctuations, with the clearest gains when residual spatial correlation was sufficiently strong. The generative leadfield model further improved sensor-space inference by avoiding inverse-first source reconstruction, including settings in which covariance modeling alone offered limited gains. Resting-state EEG validation showed higher residual correlation during eyes-closed than eyes-open rest, consistent with elevated and spatially coherent alpha activity. Applied further to propofol sedation EEG (alpha-band amplitude envelopes, 8–13 Hz), the fitted 6-node default mode network (DMN) model showed pipeline-dependent condition effects in residual spatial correlation and basin entropy, not apparent under the conventional demean-based pMEM.

## 1 Introduction

Neural activity unfolds over time through complex, nonlinear interactions among distributed brain regions. These interactions give rise to multistability, metastability, critical fluctuations, and attractor-like transitions across large-scale networks (Breakspear, 2017; Hopfield, 1982; Rabinovich et al., 2008). Empirical evidence from electrophysiology, calcium imaging, and functional MRI (fMRI) demonstrates that the brain spontaneously switches among transiently stable configurations, suggesting that large-scale neural dynamics can be understood as movement across structured energy landscapes embedded in high-dimensional state space (Haimovici et al., 2013; Kang et al., 2017, 2019; Kelso, 2012). Understanding these landscapes is fundamental for explaining how the brain maintains functional states, transitions between them, and adapts to cognitive or environmental demands.

Pairwise maximum entropy models (pMEMs) are gaining increasing attention as a principled framework for studying such nonlinear brain-state transitions. By representing each brain region as a binary active/inactive state and estimating the least-biased probability distribution over all resulting network configurations, pMEMs reconstruct an energy landscape whose local minima correspond to stable or metastable brain states (Ezaki et al., 2017; Schneidman et al., 2006; Watanabe et al., 2014). Transitions among these states can then be interpreted as movement across attractor basins, allowing the geometry of the landscape to be related to functional organization, individual differences, and changes in brain state. Previous applications have revealed attractor structure, basin geometry, and state-transition organization in cortical and subcortical networks (Kang et al., 2017, 2019).

Despite these advantages, conventional pMEM estimation faces a fundamental mismatch: neuroimaging recordings are continuous, whereas pMEM is defined over binary activation states. The standard solution is to demean each channel and hard-threshold it at zero before fitting the pMEM. Although simple, this procedure is statistically ad hoc. It discards amplitude information, ignores node-specific signal scaling, loses the sign and scale of regional responses, and produces binary time series that inherit spatial correlations present in the original measurements. Thus, the conventional pipeline can convert observation-level structure into apparent state-level coupling.

This problem has two distinct sources. First, neuroimaging measurements are not spatially independent. In EEG, for example, volume conduction induces substantial dependence among sensors even in the absence of true functional coupling, inflating apparent cross-sensor association measures such as coherence (Nunez et al., 1997; Srinivasan et al., 2007). When such correlated signals are binarized, pMEM estimation can conflate measurement artifact with genuine neural interaction: the coupling matrix J absorbs spurious co-activations driven by residual covariance, distorting the inferred energy landscape. Second, for EEG and MEG, sensor recordings reflect a linear superposition of source activity through the electromagnetic leadfield. Inverting this mixture to obtain source estimates before thresholding introduces source-superposition error, and subsequent binarization propagates this error into the inferred binary state sequence.

Recent Bayesian and empirical Bayes approaches have improved pMEM estimation by stabilizing individual-level parameter recovery and capturing subject-specific attractor organization (Jeong et al., 2021; Kang et al., 2021). However, these approaches still operate on already binarized observations and therefore do not address the more fundamental observation-model problem: how to infer latent binary network states, and their pMEM energy landscape, directly from continuous and spatially mixed neuroimaging data.

We, therefore, introduce a hierarchical generative model that embeds pMEM within a continuous observation model. Latent binary states are defined at cortical source or region-of-interest nodes and are estimated jointly with node-specific baseline and gain parameters $\left(\beta_{0},\beta_{1}\right)$, structured residual covariance, and, for EEG/MEG, a leadfield-informed forward model. In this formulation, the electromagnetic leadfield enters the likelihood as part of the generative model rather than as a preprocessing inverse, and no fixed binarization threshold is required. Crucially, the model is defined at the source level: binary states z(t) represent activity at cortical source nodes, and sensor observations are generated from these source states through the electromagnetic forward model. Source-level binary states are then recovered by inverting this generative model from sensor observations via expectation–maximization (EM) inference, without requiring an explicit source reconstruction step as preprocessing.

From this perspective, the conventional demean-and-threshold pipeline is a restrictive limiting case. It fixes the baseline $\beta_{0}$ to the empirical channel mean, leaves the gain $\beta_{1}$ unmodeled, ignores or diagonalizes residual covariance, and replaces posterior inference over z(t) with deterministic thresholding. Binarization is, therefore, reframed as an inference problem.

Figure 1 summarizes this distinction between thresholding as preprocessing and binarization as probabilistic inference within the proposed observation model.

**Fig. 1.**
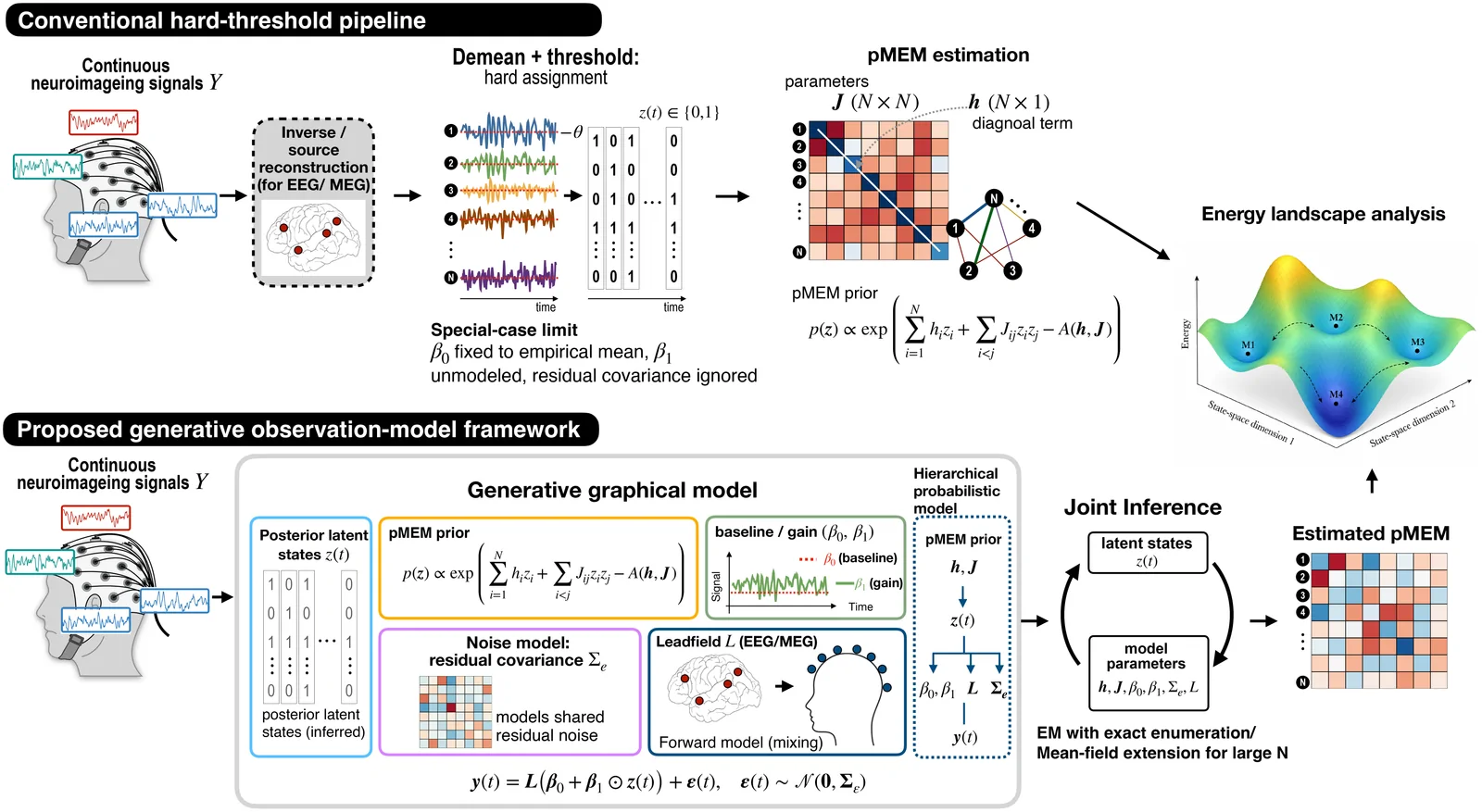
Conceptual comparison between conventional hard-threshold pMEM estimation and the proposed generative observation-model framework. The conventional pipeline converts continuous signals into binary states by demeaning and thresholding before pMEM fitting, treating binarization as a preprocessing step. This is a restrictive hard-assignment limit of the proposed model. In the proposed framework, latent binary states are inferred jointly with pMEM parameters, baseline and gain parameters, residual covariance, and the EEG/MEG leadfield. Binarization is therefore treated as probabilistic inference from continuous observations, yielding energy landscapes that account for amplitude information, shared residual structure, and source mixing.

Controlled simulations identified two complementary advantages of the proposed framework. First, modeling shared residual covariance reduced the misinterpretation of correlated measurement fluctuations as multi-node co-activation, although this benefit was regime-dependent and weakened when residual correlation was small and difficult to estimate. Second, in EEG/MEG, using the leadfield generatively reduced source-superposition error introduced by inverse-first pipelines. We evaluated these effects using synthetic data across residual-correlation levels and realistic EEG/MEG leadfields, verified stability on resting-state EEG from the PhysioNet dataset (Goldberger et al., 2000; Schalk, 2009), and illustrated practical sensitivity using 91-channel EEG recorded during propofol sedation across four experimental conditions (Chennu et al., 2016).

## 2 Methods

### 2.1 Experimental datasets and target network

We used three datasets to evaluate the proposed generative pMEM framework: two resting-state EEG datasets for real-data validation and one MEG forward-model dataset for leadfield simulation. Because the present study focuses on estimating energy landscapes from continuous neuroimaging observations, we used a small, interpretable default mode network (DMN) subnetwork as the primary target system. This choice allowed exact posterior enumeration of all binary network states and avoided unnecessary source-space dimensionality in the EEG/MEG forward model.

#### 2.1.1 DMN node definition

We selected six representative DMN nodes from the Schaefer 2018 100-parcel, 7-network cortical parcellation (Schaefer et al., 2018) on the fsaverage surface. The six-node selection was a project-specific reduction intended to approximate canonical medial and lateral DMN components while keeping the binary state space tractable for exact enumeration. The resulting network comprised medial frontal, posterior medial, bilateral inferior parietal, and bilateral temporal DMN components; the corresponding node labels, parcel assignments, and coordinates are listed in Table 1.

**Table 1.** DMN nodes used throughout this study.

Node	Hemi	Schaefer 2018	MNI (*x*, *y*, *z*)
ACC/mPFC	L/R	PFC_3, PFCdPFCm_1	1, 45, −1
PCC/precuneus	L/R	pCunPCC_1	1, −58, 9
Left IPL	L	Par_2	−42, −63, 31
Right IPL	R	Par_1	47, −55, 27
Left MTG	L	Temp_2	−49, −37, −4
Right MTG	R	Temp_3	46, −33, −3

Coordinates for bilateral midline nodes are the mean of left and right hemisphere centroids.

This 6-node DMN subnetwork was used for the EEG/MEG leadfield simulations and the real-data analyses. With *N* = 6, the complete binary state space contains 2^6^ = 64 configurations, allowing exact posterior enumeration and direct visualization of the corresponding pMEM energy landscape.

#### 2.1.2 Alternative network definitions

For the network- and frequency-band sensitivity analysis (Section 3.6), two additional 6-node networks were defined from the same Schaefer 2018 parcellation, using the same node-selection and leadfield-construction procedure as the primary DMN-6 network. The Frontoparietal Control Network (FPN-6) comprised separate left and right dorsolateral prefrontal nodes (7Networks_{LH,RH}_Cont_PFCl_1), separate left and right posterior parietal nodes (Cont_Par_1), and bilateral-averaged cingulate (Cont_Cing_1) and precuneus (Cont_pCun_1) nodes, mirroring the DMN-6 bilateral-collapse convention. The visual network (Vis-6) comprised bilateral early-occipital (Vis_1) and middle-occipital (Vis_5) node pairs, and left (Vis_9) and right (Vis_8) late-occipital nodes.

#### 2.1.3 Frequency-band selection

All primary empirical analyses used alpha-band (8–13 Hz) amplitude envelopes. Alpha was selected because it is a dominant rhythm in resting-state scalp EEG, has relatively favorable signal-to-noise characteristics in eyes-closed and sedated recordings, and has been consistently associated with arousal state and propofol-induced changes in consciousness (Chennu et al., 2016; Cimenser et al., 2011; Purdon et al., 2013). It, therefore, provided a biologically well-motivated first frequency band in which to apply the proposed observation model. The framework is not restricted to alpha: the same inference framework can be applied to other bands without methodological change. Frequency-specific residual covariance and energy-landscape findings, however, need not be identical across bands, since these reflect the biological signals present within each band rather than the estimation procedure itself. We also conducted a gamma-band analysis of the propofol sedation data, reported in Section 3.6.

#### 2.1.4 PhysioNet EEG motor movement/imagery dataset

Eyes-open and eyes-closed baseline recordings from 20 participants were selected from the PhysioNet EEG Motor Movement/Imagery repository (64-channel, 160 Hz) (Goldberger et al., 2000; Schalk, 2009). Alpha-band amplitude envelopes (8–13 Hz) were extracted via Hilbert transformation using MNE-Python (Gramfort et al., 2014), downsampled to 25 Hz, and unit-variance normalized. An EEG forward model for the six DMN nodes was computed using the fsaverage boundary element model (BEM). Because ground-truth parameters are unavailable, this dataset served as a real-data stability and interpretability check.

#### 2.1.5 MNE sample MEG dataset for leadfield simulation

To evaluate the method under realistic MEG sensor geometry, we used the MEG forward solution from the publicly available MNE-Python sample dataset (Gramfort et al., 2014) (102 magnetometer channels). Schaefer 2018 DMN-6 parcels were registered to the subject anatomy, and parcel-averaged leadfield columns were extracted to form $\text{L}\in {\mathbb{R}}^{102\times 6}$ . No empirical MEG time series were analyzed; the dataset was used solely to supply a realistic forward operator for the simulation in Section 3.

#### 2.1.6 Chennu propofol sedation EEG dataset

We used the propofol sedation EEG dataset of Chennu et al. (2016), comprising resting-state high-density EEG (91 channels) from 20 healthy adults across four conditions: baseline, mild sedation, moderate sedation, and recovery. Alpha-band amplitude envelopes (8–13 Hz) were extracted via Hilbert transformation and downsampled to 25 Hz. A subject-invariant EEG forward model for the six DMN nodes was computed using the fsaverage BEM. This dataset was used to compare the condition-dependent landscape summaries obtained from the proposed model and the conventional demean-and-threshold pipeline.

### 2.2 The state model

For a network of *N* cortical source nodes, let $z={\left(z_{1},\ldots ,z_{N}\right)}^{⊤}\in {\left\{0,1\right\}}^{N}$ denote the source-level latent binary activation pattern, where *z_i_*(*t*) = 1 indicates that source node *i* is in the active state at time *t*. We assume that z follows a pairwise maximum entropy model (pMEM): for $\text{z}\in {\left\{0,1\right\}}^{N}$

$$p\left(\text{z}\right)=\text{exp}\left(\sum_{i=1}^{N}h_{i}z_{i}+\sum_{i<j}J_{ij}z_{i}z_{j}-A\left(\text{h},\text{J}\right)\right)$$ (1)

where *h_i_* and *J_ij_* are the model parameters for pMEM, and A(h,J) is the cumulant function or the log-partition function given by

$$A\left(\text{h},\text{J}\right)=\text{log}\sum_{\text{z}\in {\left\{0,1\right\}}^{N}}\text{exp}\left(\sum_{i=1}^{N}h_{i}z_{i}+\sum_{i<j}J_{ij}z_{i}z_{j}\right)$$ (2)

with $\text{h}={\left(h_{1},h_{2},...,h_{N}\right)}^{⊤}$ and $\text{J}=\left(J_{ij}\right)$, *J_ij_* = 0 for *i* ≥ *j*.

### 2.3 The observation model

Let $\text{y}\left(t\right)={\left(y_{1}\left(t\right),\;\ldots ,y_{N}\left(t\right)\right)}^{⊤}$ be the vector of observed continuous signals at time *t*, where *y_i_*(*t*) may represent any scalar summary of node *i*’s activity, such as raw signal amplitude, a bandpass amplitude envelope, or instantaneous power.

We model the conditional distribution p(y|z) as a multivariate Gaussian:

$$\text{y}|\text{z}∼N\left(g\left(\text{z}|\beta_{0},\beta_{1}\right),{\text{P}}^{-1}\right)$$ (3)

Here, $g(\text{z}|\beta_{0},\;\beta_{1})=\beta_{0}+\beta_{1}⊙\text{z}$ is the mean vector for the observed signals, where $\beta_{0}={\left(\beta_{01},\;\beta_{02},\;...,\;\beta_{0N}\right)}^{⊤}$ and $\beta_{1}={\left(\beta_{11},\;\beta_{12},\;...,\;\beta_{1N}\right)}^{⊤}$ are the coefficient vectors of length *N*, and ⊙ is the Hadamard product. The parameter β_0_*_i_* is the expected signal level at node *i* in the inactive state, and β_1_*_i_* is the additional gain on top of this baseline when node *i* is active. In the primary empirical analyses presented here, *y_i_*(*t*) is the alpha-band amplitude envelope (8–13 Hz, Hilbert-extracted); the binary state *z_i_*(*t*) = 1 therefore denotes a high-alpha epoch at source node *i*.

Throughout, we adopt the labeling convention β_1_*_i_* > 0, so that *z_i_*(*t*) = 1 denotes the higher-amplitude state at each node; this convention fixes the node-wise relabeling invariance of the model and makes the estimated fields and couplings directly comparable across nodes, subjects, and conditions.

The precision matrix $\text{P}=\left(p_{ij}\right)$ is parametrized as a linear combination of known symmetric basis matrices ${\text{Q}}_{k}$:

$$\text{P}=\sum_{k=1}^{K}\;\lambda_{k}{\text{Q}}_{k},$$

with coefficients λ*_k_* constrained so that P is positive definite.

In the present implementation, we use *K* = 2, $Q_{1}\;=\text{I}$ , and $Q_{2}\;=11^{⊤}$, yielding the compound-symmetry precision

$$\text{P}=\lambda_{1}\text{I}+\lambda_{2}\text{1}{\text{1}}^{⊤}.$$

This parsimonious structure provides a first-order correction for shared residual variance.

Let

$$\text{Y}={\left[\text{y}\left(1\right),\;\text{y}\left(2\right),\;\cdots ,\;\text{y}\left(T\right)\right]}^{⊤}$$

and

$$\text{Z}={\left[\text{z}\left(1\right),\text{z}\left(2\right),\cdots ,\text{z}\left(T\right)\right]}^{⊤}$$

be matrices, where y(t) is the observed signal vector and z(t) is the (realized) latent variable at time t=1,2,...,T , respectively. Then, assuming temporal independence of the observed signals given the latent variables, the log-likelihood of the observed data is expressed as:

$$\begin{array}{c} \begin{array}{l} \log p\text{(}Y|Z\text{)}\\ \;=-\frac{TN}{2}\text{log}2\pi +\frac{T}{2}\text{logdet}\left(\text{P}\right)-\frac{1}{2}\sum_{t=1}^{T}\text{r}{\left(t\right)}^{⊤}\text{Pr}\left(t\right) \end{array} \end{array}$$ (4)

with $\text{r}\left(t\right)=\text{y}\left(t\right)-\beta_{0}\;-\beta_{1}⊙\text{z}\left(t\right)$, t=1,2, ...,T .

#### 2.3.1 Relation to demean-and-threshold pMEM

The conventional pMEM preprocessing pipeline can be viewed as a restrictive hard-assignment limit of the present observation model. Demeaning each channel and thresholding at zero gives

$$z_{i}^{\text{thr}}\left(t\right)=I\left\{y_{i}\left(t\right)-{\bar{y}}_{i}>0\right\},\;\;{\bar{y}}_{i}=\frac{1}{T}\sum_{t=1}^{T}\;y_{i}\left(t\right).$$

Under the observation model, ${\bar{y}}_{i}\;\approx \beta_{0i}\;+\beta_{1i}\;\text{Pr}\left(z_{i}=1\right)$. Conventional thresholding therefore fixes the baseline from preprocessing, does not estimate the gain β_1_*_i_*, ignores residual covariance, replaces the posterior p(z(t)|y(t)) with a hard point estimate, and estimates J only after binary states have already been assigned. The proposed model relaxes these restrictions by estimating $\beta_{0}$, $\beta_{1}$, residual precision, latent-state posterior moments, and pMEM parameters jointly.

### 2.4 Posterior

Let $\theta =\left(\beta_{0},\;\;\beta_{1},\;\;\lambda ,\;\;\text{h},\;\;\text{J}\right)$ collect all model parameters. The log-posterior of the latent states decomposes as

$$\begin{array}{c} \begin{array}{l} \text{log}\;p\left(\text{Z}|\text{Y},\theta \right)\\ \;=\text{log}\;p\left(\text{Z}|\theta \right)+log\;p\left(\text{Y}|\text{Z},\theta \right)-log\;p\left(\text{Y}|\theta \right). \end{array} \end{array}$$ (5)

The Gaussian observation likelihood in Eq. (4) contributes a quadratic form in z(t). Using the binary identity *z_i_*(*t*)^2^ = *z_i_*(*t*), the diagonal quadratic terms can be absorbed into the fields. The posterior, therefore, factorizes over time into time-specific pMEM forms; equivalently, its log-density can be written, up to an additive constant, as

$$\begin{array}{c} \begin{array}{l} \text{log}\;p\left(\text{Z}|\text{Y},\theta \right)\\ \;=\sum_{t=1}^{T}\left[\sum_{i=1}^{N}\;{\tilde{h}}_{i}\left(t\right)z_{i}\left(t\right)+\sum_{i<j}{\tilde{J}}_{ij}z_{i}\left(t\right)z_{j}\left(t\right)-A\left(\tilde{\text{h}}\left(t\right),\tilde{\text{J}}\right)\right] \end{array} \end{array}$$

with

$${\tilde{h}}_{i}\left(t\right)=h_{i}+\beta_{1i}\sum_{i^{\prime }=1}^{N}p_{ii^{\prime }}\left(y_{i^{\prime }}\left(t\right)-\beta_{0i^{\prime }}\right)-\frac{1}{2}p_{ii}\beta_{1i}^{2}$$ (6)

for i=1,2, ..., N;

$${\tilde{J}}_{ij}=\{\begin{array}{ll} J_{ij}-\beta_{1i}\beta_{1j}p_{ij} & \text{for }1\leq i<j\leq N\\ 0 & \text{otherwise} \end{array}$$ (7)

### 2.5 The EM algorithm

We estimate the model parameters $\theta =\left(\beta_{0},\beta_{1},\lambda ,\text{h},\text{J}\right)$ by expectation–maximization (EM). The E-step forms the expected complete-data log-likelihood under the current posterior over the latent states

$$Q\left(\theta |\theta^{\text{old}}\right)=\sum_{t=1}^{T}\;E\left[\text{log}\;p\left(\text{z}\left(t\right),\text{y}\left(t\right)|\theta \right)|\text{Y},\theta^{\text{old}}\right],$$

and the M-step maximizes *Q* over θ. The posterior p(z(t)|y(t),θ) is the time-varying pMEM with parameters in Eqs. (6)–(7).

The following E- and M-steps were alternated for a fixed iteration budget while monitoring the objective and parameter trajectories:

*E-step.* For each *t*, enumerate the posterior moments

$$\text{m}\left(t\right)=\left(m_{i}\left(t\right)\right)=E\left[\text{z}\left(t\right)|\text{Y}\right],$$ (8)

$$\text{C}\left(t\right)=\left(C_{ij}\left(t\right)\right)=E\left[\text{z}\left(t\right)\text{z}{\left(t\right)}^{⊤}|\text{Y}\right].$$ (9)

Note that *C_ii_*(*t*) = *m_i_*(*t*) holds for i=1, 2, ..., N by the binary identity *z_i_*(*t*)^2^ = *z_i_*(*t*).

*M-step.* Using the moment identities in Eqs. (8)–(9), the expected complete-data log-likelihood is, up to a constant in θ,

$$\begin{array}{l} Q\left(\theta |\theta^{\text{old}}\right)\\ \;=\sum_{t=1}^{T}\left\{\sum_{i}\;h_{i}m_{i}\left(t\right)+\sum_{i<j}\;J_{ij}C_{ij}\left(t\right)\right\}-T\cdot A\left(\text{h},\text{J}\right)\\ \;-\frac{1}{2}\sum_{t=1}^{T}\bar{\text{r}}{\left(t\right)}^{⊤}\text{P}\bar{\text{r}}\left(t\right)-\frac{1}{2}\sum_{t=1}^{T}\;\beta_{1}^{⊤}\left(\text{P}⊙\text{Cov}\left(\text{z}\left(t\right)|\text{Y}\right)\right)\beta_{1}\\ \;+\frac{T}{2}\text{log det }\text{P}, \end{array}$$

with $\bar{\text{r}}\left(t\right)=\text{y}\left(t\right)-\beta_{0}\;-\beta_{1}⊙\text{m}\left(t\right)$ and Cov(z(t)|Y)=C(t)− $\text{m}\left(t\right)\text{m}{\left(t\right)}^{⊤}$. The variance-correction term $\beta_{1}^{T}(P⊙Cov(z(t)|Y))\beta_{1}$ arises from $E[{\left(\beta_{1}⊙\text{z}\right)}^{⊤}\text{P}\left(\beta_{1}⊙\text{z}\right)|\text{Y},\theta ]$ and accounts for posterior uncertainty in the latent states.

1. (Update h and J) The gradient of *Q* involves the prior moments

$$m_{i}^{\left(0\right)}=E_{\text{z}|\text{h},\text{J}}\left[z_{i}\right],\;m_{ij}^{\left(0\right)}=E_{\text{z}|\text{h},\text{J}}\left[z_{i}z_{j}\right]$$

for i, j=1, 2, ..., N . Then, we take gradient-ascent steps

$$\begin{array}{l} h_{i}\leftarrow h_{i}+\eta_{h}\left[\frac{1}{T}\sum_{t}m_{i}\left(t\right)-m_{i}^{\left(0\right)}\right]\\ \;\;\;\text{for }1\leq i\leq N; \end{array}$$ (10)

$$\begin{array}{l} J_{ij}\leftarrow J_{ij}+\eta_{J}\left[\frac{1}{T}\sum_{t}C_{ij}\left(t\right)-m_{ij}^{\left(0\right)}\right]\\ \;\;\;\text{for }1\leq i<j\leq N \end{array}$$ (11)

2. (Update $\beta_{0}$ and $\beta_{1}$) Unlike (h,J) and the noise parameters, $\beta_{0}$ and $\beta_{1}$ have closed-form M-step updates. Setting $\partial Q/\partial \beta_{0}\;=0$ ,

$$\beta_{0}\;=\frac{1}{T}\sum_{t=1}^{T}\;\left(\text{y}\left(t\right)-\beta_{1}⊙\text{m}\left(t\right)\right),$$ (12)

and setting $\partial Q/\partial \beta_{1}=0$ gives the linear equation system $\text{A}\beta_{1}=\text{b}$ with

$$\text{A}=\sum_{t=1}^{T}\;\text{P}⊙\text{C}\left(t\right)$$ (13)

and

$$\text{b}=\sum_{t=1}^{T}\;\text{m}\left(t\right)⊙\text{P}\left(\text{y}\left(t\right)-\beta_{0}\right).$$ (14)

3. (Update the noise parameters) For k=1, …, K ,

$$\begin{array}{l} \frac{\partial Q}{\partial \lambda_{k}}=\frac{T}{2}\text{tr}\left({\text{P}}^{-1}{\text{Q}}_{k}\right)-\frac{1}{2}\sum_{t=1}^{T}\bar{\text{r}}{\left(t\right)}^{⊤}{\text{Q}}_{k}\bar{\text{r}}\left(t\right)\\ \;\;\;\;-\frac{1}{2}\sum_{t=1}^{T}\;\beta_{1}^{⊤}\left({\text{Q}}_{k}⊙\text{Cov}\left[\text{z}\left(t\right)|\text{Y}\right]\right)\beta_{1}. \end{array}$$ (15)

We, therefore, take gradient-ascent steps

$$\begin{array}{l} \begin{array}{l} \lambda_{k}\;\leftarrow \lambda_{k}\;+\eta_{\lambda }\{\text{tr}\left({\text{P}}^{-1}{\text{Q}}_{k}\right)-\frac{1}{T}\sum_{t=1}^{T}\;\bar{\text{r}}{\left(t\right)}^{⊤}{\text{Q}}_{k}\bar{\text{r}}\left(t\right)\\ \;\;\;\;\;\;-\frac{1}{T}\sum_{t=1}^{T}\;\beta_{1}^{⊤}\left({\text{Q}}_{k}⊙\text{Cov}\left[\text{z}\left(t\right)|\text{Y}\right]\right)\beta_{1}\} \end{array} \end{array}$$ (16)

with learning rate η_λ_ > 0, projecting onto P≻0 after each step (e.g., by clipping the eigenvalues of P).

#### 2.5.1 Scalable extension for large networks

For larger networks, exact enumeration of the 2*^ N^* states becomes intractable. Because the model assumes conditional independence across time points given θ, the posterior already factorizes exactly over time, p(Z|Y,θ) $=\prod_{t}p\left(\text{z}\left(t\right)|\text{y}\left(t\right),\theta \right)$; the approximation required for large *N* is therefore applied only across nodes *within* each time point, replacing the exact per-time-point posterior with the fully factorized mean-field form

$$q_{t}\left(\text{z}\left(t\right)\right)=\underset{i}{\prod }\;\text{Bernoulli}\left(m_{i}\left(t\right)\right),$$

yielding a variational EM extension. For a fixed time point, the unnormalized log-posterior has the form $\sum_{i}{\tilde{h}}_{i}\left(t\right)z_{i}\left(t\right)+\sum_{i<j}\;{\tilde{J}}_{ij}z_{i}\left(t\right)z_{j}\left(t\right)$ (Eqs. (6)–(7)), which already incorporates the observation likelihood through ${\tilde{h}}_{i}\left(t\right)$ and ${\tilde{J}}_{ij}$ . The per-time-point posterior is therefore itself a pairwise MEM, and maximizing the corresponding mean-field variational free energy with respect to *m_i_*(*t*), holding the other node-wise means fixed, gives the naive mean-field fixed-point iteration (Welling & Hinton, 2002): for i=1, …,N

$$m_{i}\left(t\right)\leftarrow \sigma \left({\tilde{h}}_{i}\left(t\right)+\sum_{j<i}\;{\tilde{J}}_{ji}m_{j}\left(t\right)+\sum_{j>i}\;{\tilde{J}}_{ij}m_{j}\left(t\right)\right),$$ (17)

where $\sigma \left(x\right)=1/\left(1+e^{-x}\right)$ is the logistic sigmoid and the two sums follow from the upper-triangular convention *J_ij_* = 0 for *i* ≥ *j* in Eq. (1). The mean-field approximation sets *C_ii_*(*t*) = *m_i_*(*t*) and *C_ij_*(*t*) = *m_i_*(*t*)*m_j_*(*t*) for *i* ≠ *j*, and hence

$${\text{Cov}}_{q}\left[\text{z}\left(t\right)\right]=\text{diag}\left(\text{m}\left(t\right)⊙\left(1-\text{m}\left(t\right)\right)\right).$$

Substituting these into the M-step formulas above yields the variational updates. This node-wise factorization neglects posterior dependencies between nodes at a given time point, so it is best understood as a computationally scalable first-order approximation: it does not represent correlated posterior uncertainty and may be less accurate for strongly coupled or multimodal posteriors.

#### 2.5.2 Initialization and convergence

Model parameters were initialized using data-driven estimates. The field parameters were initialized as h=0 . The coupling matrix J was initialized with small symmetric random values drawn from $N\left(0,\;0.01^{2}\right)$; this perturbation is far smaller than the fitted coupling magnitudes and serves only to avoid starting from a perfectly symmetric zero-coupling configuration.

An initial latent-state estimate ${\text{m}}^{\left(0\right)}$ was obtained by thresholding the observed signal at its per-channel mean for the direct-observation model, or the reconstructed source signal at its per-channel median for the leadfield model. $\beta_{0}$ and $\beta_{1}$ were then estimated by regressing the corresponding channel-level or reconstructed source-level signal onto ${\text{m}}^{\left(0\right)}$. Independently of this, the residual-covariance parameters were initialized from a proxy residual computed directly from the raw signal (the covariance of the demeaned signal for the direct-observation model; a source-subtracted residual for the leadfield model, Section 2.6). Both sets of parameters were subsequently refined by the M-step at each EM iteration. To maintain a consistent definition of the active state, the sign of $\beta_{1}$ was constrained to be positive by relabeling the corresponding latent states when necessary.

The objective is non-convex and global optimality is not guaranteed; we, therefore, monitored the objective and parameter trajectories throughout fitting. We used 200 EM iterations for the direct-observation simulations, the fixed budget applied consistently across all such analyses. For the more complex leadfield simulations and empirical EEG analyses, we used 600 iterations based on the convergence assessment described below. This budget was selected from a convergence check across budgets of 150–1500 iterations on five subjects each from the propofol and PhysioNet datasets: the fitted noise parameters (σ^2^, ρ) changed by at most 0.02% (propofol) and 0.01% (PhysioNet), and the EM objective by at most 0.06% and 0.14% respectively, between 600 iterations and the longest budget tested, confirming that 600 iterations was sufficient for stabilization of the monitored objective and noise parameters in the datasets examined; this check does not by itself establish the stability of (h,J) or the discrete landscape summaries *n_min_* and $H_{\text{basin}}$ at this budget, which are addressed separately by the restart-sensitivity analysis below.

### 2.6 EEG leadfield observation model

When sensor-level EEG/MEG data are available, the observation model is extended to include the electromagnetic forward operator, enabling direct inference of source-level binary states z(t) from sensor observations without an explicit source reconstruction preprocessing step. Let $\text{L}\in {\mathbb{R}}^{M\times N}$ be the leadfield matrix, where *M* is the number of sensors and *N* is the number of cortical source ROIs. The sensor-space observation model is

$$\text{y}\left(t\right)=\text{L}\left(\beta_{0}+\beta_{1}⊙\text{z}\left(t\right)\right)+\varepsilon \left(t\right),\;\varepsilon \left(t\right)\sim N\left(0,\Sigma_{\varepsilon }\right)$$ (18)

Here, $\text{s}\left(t\right)=\beta_{0}+\beta_{1}⊙\text{z}\left(t\right)$ is the source signal vector: β_0_*_i_* is the expected signal level at source *i* in the inactive state; and β_1_*_i_* is the additional level when *z_i_*(*t*) = 1. The leadfield L maps these source-level signals to sensor-space observations. When y(t) is a bandpass amplitude envelope, L functions as an effective linear mixing operator rather than an exact instantaneous voltage forward model, because envelope extraction is nonlinear. $\varepsilon \left(t\right)\sim N\left(0,\;\Sigma_{\varepsilon }\right)$ is the residual sensor noise with compound-symmetry structure,

$$\Sigma_{\varepsilon }\;=\sigma^{2}\left[\left(1-\rho \right)\text{I}+\rho \text{1}{\text{1}}^{⊤}\right],$$

capturing shared residual covariance after the modeled source contribution has been removed. Unlike the direct ROI model, the precision matrix ***P$:=\Sigma_{\varepsilon }^{-1}\in {\mathbb{R}}^{M\times M}$*** is defined in sensor space, and the corresponding source-projected precision entering the latent-state posterior is ${\text{L}}^{⊤}\text{PL}\in {\mathbb{R}}^{N\times N}$ .

The leadfield model preserves the same latent pMEM prior, but the observation likelihood enters the latent-state posterior through the source-projected precision

$$\text{K}\;={\text{L}}^{⊤}\text{PL}\in {\mathbb{R}}^{N\times N}.$$

The posterior over z(t) therefore retains the pMEM form, with the observation-driven linear and pairwise terms computed using ${\text{L}}^{⊤}\text{P}$ and K, respectively. We write

$$\bar{\text{r}}\left(t\right)=\text{y}\left(t\right)-\text{L}\left(\beta_{0}+\beta_{1}⊙\text{m}\left(t\right)\right)$$

for the sensor-space residual mean and

$$\bar{\text{m}}=T^{-1}\sum_{t}\text{m}\left(t\right).$$

The $\beta_{0}$ update becomes a Tikhonov regularized weighted least-squares problem in source space,

$$\beta_{0}\;={\left({\text{L}}^{⊤}\text{PL}+\mu \text{I}\right)}^{-1}{\text{L}}^{⊤}\text{P}{\bar{\text{r}}}_{0},$$ (19)

where ${\bar{\text{r}}}_{0}=T^{-1}\sum_{t}\left\{\text{y}\left(t\right)-\text{L}\left(\beta_{1}⊙\bar{\text{m}}\right)\right\}$ distinct from the per-time residual $\bar{\text{r}}\left(t\right)$. After globally standardizing y(t) across channels and time, we fixed $\mu =10^{-3}$ in all leadfield analyses to stabilize ill-conditioned directions of ${\text{L}}^{⊤}\text{PL}$ ; preliminary simulations showed stable recovery across smaller values. The $\beta_{0}$ update is unaffected by the variance correction because it enters $\bar{\text{r}}\left(t\right)$ linearly.

The $\beta_{1}$ update solves the *N*×*N* linear system $\text{A}\beta_{1}=\text{b}$ with

$$\begin{array}{l} \text{A}=\sum_{t=1}^{T}\text{K}⊙\text{C}\left(t\right),\\ \text{b}=\sum_{t=1}^{T}\text{m}\left(t\right)⊙{\text{L}}^{⊤}\text{P}\left(\text{y}\left(t\right)-\text{L}\beta_{0}\right), \end{array}$$

where $\text{C}\left(t\right)=E_{q}\left[\text{z}\left(t\right)\text{z}{\left(t\right)}^{⊤}\right]$ as defined above.

For the noise hyperparameters, the log det P term and the residual-quadratic term are evaluated in sensor space and therefore retain the sensor-space basis ${\text{Q}}_{k}\in {\mathbb{R}}^{M\times M}$ unchanged; only the source-space variance-correction term involves the projected basis ${\text{L}}^{⊤}{\text{Q}}_{k}\text{L}$ . The gradient is

$$\begin{array}{l} \frac{\partial Q}{\partial \lambda_{k}}=\frac{T}{2}\text{tr}\left({\text{P}}^{-1}{\text{Q}}_{k}\right)-\frac{1}{2}\sum_{t=1}^{T}\bar{\text{r}}{\left(t\right)}^{⊤}{\text{Q}}_{k}\bar{\text{r}}\left(t\right)\\ \;\;\;-\frac{1}{2}\sum_{t=1}^{T}\;\beta_{1}^{⊤}\left(\left({\text{L}}^{⊤}{\text{Q}}_{k}\text{L}\right)⊙\text{Cov}\left[\text{z}\left(t\right)|\text{Y}\right]\right)\beta_{1}, \end{array}$$ (20)

where $\bar{\text{r}}\left(t\right)=\text{y}\left(t\right)-\text{L}\left(\beta_{0}+\beta_{1}⊙\text{m}\left(t\right)\right)\in {\mathbb{R}}^{M}$ is the sensor-space residual mean and $\text{P}\in {\mathbb{R}}^{M\times M}$ . The source-space variance-correction term admits the equivalent sensor-space form

$$\begin{array}{l} \beta_{1}^{⊤}\left(\left({\text{L}}^{⊤}{\text{Q}}_{k}\text{L}\right)⊙\text{Cov}\left[\text{z}\left(t\right)|\text{Y}\right]\right)\beta_{1}\\ \;=\text{tr}\left(\text{L}\text{diag}\left(\beta_{1}\right)\text{Cov}\left[\text{z}\left(t\right)|\text{Y}\right]\text{diag}\left(\beta_{1}\right){\text{L}}^{⊤}{\text{Q}}_{k}\right), \end{array}$$ (21)

so that, as in the direct-observation model, every term is ultimately contracted against the sensor-space basis ${\text{Q}}_{k}$. The corresponding gradient-ascent update is

$$\begin{array}{l} \lambda_{k}\leftarrow \lambda_{k}+\eta_{\lambda }\{\text{tr}\left({\text{P}}^{-1}{\text{Q}}_{k}\right)-\frac{1}{T}\sum_{t=1}^{T}\bar{\text{r}}{\left(t\right)}^{⊤}{\text{Q}}_{k}\bar{\text{r}}\left(t\right)\\ \;\;\;\;-\frac{1}{T}\sum_{t=1}^{T}\;\beta_{1}^{⊤}\left(\left({\text{L}}^{⊤}{\text{Q}}_{k}\text{L}\right)⊙\text{Cov}\left[\text{z}\left(t\right)|\text{Y}\right]\right)\beta_{1}\} \end{array}$$ (22)

with the same projection onto P≻0 (via the eigenvalues of P) used in the direct-observation model.

Note that, for the compound-symmetry noise model used throughout, the precision is spanned by the two-component basis ${\text{Q}}_{1}\;=\text{I}$ and ${\text{Q}}_{2}=11^{⊤}$, so that $\text{P}=\lambda_{1}\text{I}+\lambda_{2}\text{1}{\text{1}}^{⊤}$ is itself of compound-symmetry form. The fitted coefficients (λ_1_, λ_2_) determine the noise parameters (σ^2^, ρ) in closed form by inverting

$$\lambda_{1}=\frac{1}{\sigma^{2}\left(1-\rho \right)},\;\lambda_{1}+M\lambda_{2}=\frac{1}{\sigma^{2}\left(1-\rho +M\rho \right)},$$ (23)

which are the two distinct eigenvalues of P (with multiplicities *M*−1 and 1, respectively). Since ρ > 0 corresponds to λ_2_ < 0, the positivity constraint is imposed on the eigenvalues of P (namely λ_1_ > 0 and $\lambda_{1}+\text{M}\lambda_{2}>0$ ) rather than on λ_2_ itself, ensuring that P is positive definite. In practice, (σ^2^, ρ) are initialized from the covariance of signal-subtracted residuals (a regularized pseudoinverse of L applied to raw Y removes the bulk of the source signal before the compound-symmetry fit) and refined each M-step from the model residuals $\bar{\text{r}}\left(t\right)$.

Physically, ρ captures residual inter-sensor covariance *after* the modeled source activity $\text{L}\left(\beta_{0}+\beta_{1}⊙\text{z}\right)$ has been subtracted; it reflects unmodeled cortical sources, imperfect forward modeling, residual artifacts, and referencing effects. When $\hat{\rho }$ is small (e.g., after aggressive artifact rejection), the method reduces gracefully toward the diagonal-noise limit without requiring model re-specification.

#### 2.6.1 Implementation

In the implementation used throughout this paper, gradient ascent on the λ_k_ is replaced by closed-form moment-matching. For the compound-symmetry parameterization $\Sigma_{\varepsilon }=\sigma^{2}\left[\left(1-\rho \right)\text{I}+\rho \text{1}{\text{1}}^{⊤}\right]$, setting $\partial Q/\partial \lambda_{k}\;=0$ analytically gives

$${\hat{\sigma }}^{2}=D^{-1}\text{tr}\left(\bar{\text{S}}\right),$$ (24)

$$\hat{\rho }={\bar{s}}_{\text{off}}/{\hat{\sigma }}^{2},$$ (25)

where *D* is the dimensionality of $\bar{\text{S}}$: *D* = *N* for the direct-observation model, and *D* = *M* for the leadfield model (Section 2.6). Writing

$${\bar{\text{Cov}}}_{q}\left[\text{z}\right]=\frac{1}{T}\sum_{t=1}^{T}{\text{Cov}}_{q}\left[\text{z}\left(t\right)\right]$$

for the time-averaged posterior covariance, the residual second-moment matrix $\bar{\text{S}}$ is, for the direct-observation model,

$$\begin{array}{c} \bar{\text{S}}=\frac{1}{T}\sum_{t}\;\bar{\text{r}}\left(t\right)\bar{\text{r}}{\left(t\right)}^{⊤}+\text{diag}\left(\beta_{1}\right){\bar{\text{Cov}}}_{q}\left[\text{z}\right]\text{diag}\left(\beta_{1}\right), \end{array}$$ (26)

and for the leadfield model,

$$\begin{array}{c} \bar{\text{S}}=\frac{1}{T}\sum_{t}\;\bar{\text{r}}\left(t\right)\bar{\text{r}}{\left(t\right)}^{⊤}\;+\text{L}\;\text{diag}\left(\beta_{1}\right){\bar{\text{Cov}}}_{q}\left[\text{z}\right]\;\text{diag}\left(\beta_{1}\right){\text{L}}^{⊤}, \end{array}$$ (27)

in both cases the moment-matching estimate of the residual covariance, since $E_{q}[\left(\text{y}-\mu \left(\text{z}\right)\right){\left(\text{y}-\mu \left(\text{z}\right)\right)}^{⊤}]=$ $\bar{\text{r}}{\bar{\text{r}}}^{⊤}+\text{diag}\left(\beta_{1}\right){\text{Cov}}_{q}\left[\text{z}\right]\text{diag}\left(\beta_{1}\right)$ (with L inserted for the leadfield case). ${\bar{s}}_{\text{off}}$ denotes the mean off-diagonal entry of $\bar{\text{S}}$. For numerical stability, the closed-form estimates are incorporated via an exponential moving average (EMA):

$$\sigma^{2}\leftarrow \left(1-\alpha \right)\sigma^{2}+\alpha {\hat{\sigma }}^{2},$$ (28)

$$\rho \leftarrow \left(1-\alpha \right)\rho +\alpha \hat{\rho },$$ (29)

The EMA parameter α determines how rapidly the noise-parameter estimates are updated across EM iterations. We used α=0.3 for the direct-observation model and α=0.5 for the leadfield model because these values empirically provided stable convergence in the respective implementations; they are numerical stabilization settings rather than physiologically meaningful parameters. All empirical results reported in this paper use the EMA update; the smoothing dampens oscillations in the noise estimates during early EM iterations. Within the tested range and datasets (five propofol subjects, α∈{0.3,0.5,1.0}), fits obtained with α=0.3 and α=1.0 correlated with the α=0.5 fit at r>0.99999 at the 600-iteration budget, indicating that α did not materially affect the fitted continuous-parameter solution in the settings examined here. We separately compared each α’s fit at a shorter budget (300 iterations) against its own 600-iteration fit, to isolate residual budget-sensitivity from the choice of α: these within-α correlations ranged r=0.979 –0.992 (mean 0.988) and were essentially identical across α (mean 0.9883, 0.9884, and 0.9884 for α=0.3 , 0.5, and 1.0 respectively), that is, we found no material effect of α on the converged solution or the residual budget sensitivity in the tested settings (five propofol subjects, one condition, α∈{0.3,0.5,1.0}).

### 2.7 Energy-landscape characterization

A central goal of pMEM estimation is to characterize the energy landscape of neural state space and its attractor structure. For a fitted pMEM with parameters (h,J), the energy of state z is

$$E\left(\text{z}\right)=-{\text{h}}^{⊤}\text{z}-{\text{z}}^{⊤}\text{Jz},$$

so that $p\left(\text{z}\right)\propto e^{-E\left(\text{z}\right)}$. For networks small enough to enumerate exhaustively (e.g., *N* = 6 nodes, 2^6^ = 64 states), a state is classified as a *local minimum* if all states differing from it by a single-node flip have higher energy, and each state is assigned to the basin of the local minimum reached by iteratively moving to its lowest-energy single-flip neighbor.

Two scalar summaries characterize the resulting landscape. The *number of local minima n_min_* counts the attractor states; larger values indicate a more fragmented landscape. *Basin entropy* $H_{\text{basin}}$ is the Shannon entropy of the Boltzmann probability mass distributed across attractor basins:

$$H_{\text{basin}}=-\sum_{k=1}^{n_{\text{min}}}\;p_{k}\text{log}\;p_{k},\;p_{k}=\frac{\sum_{s\in {ℬ}_{k}}e^{-E\left(s\right)/T_{B}}}{\sum_{s}e^{-E\left(s\right)/T_{B}}},$$

where ${ℬ}_{k}$ is the set of states in basin *k* and *T_B_* = 1 (Boltzmann temperature). Higher $H_{\text{basin}}$ indicates more evenly distributed occupancy across multiple attractors; lower values reflect concentration of probability mass in a single dominant basin. $H_{\text{basin}}$ captures landscape fragmentation more richly than *n_min_* alone: two landscapes may share the same number of attractors yet differ dramatically in basin occupancies.

For illustration, Figure 2 applies these definitions to a fixed, hand-chosen synthetic six-node pMEM (not empirically fitted):

**Fig. 2.**
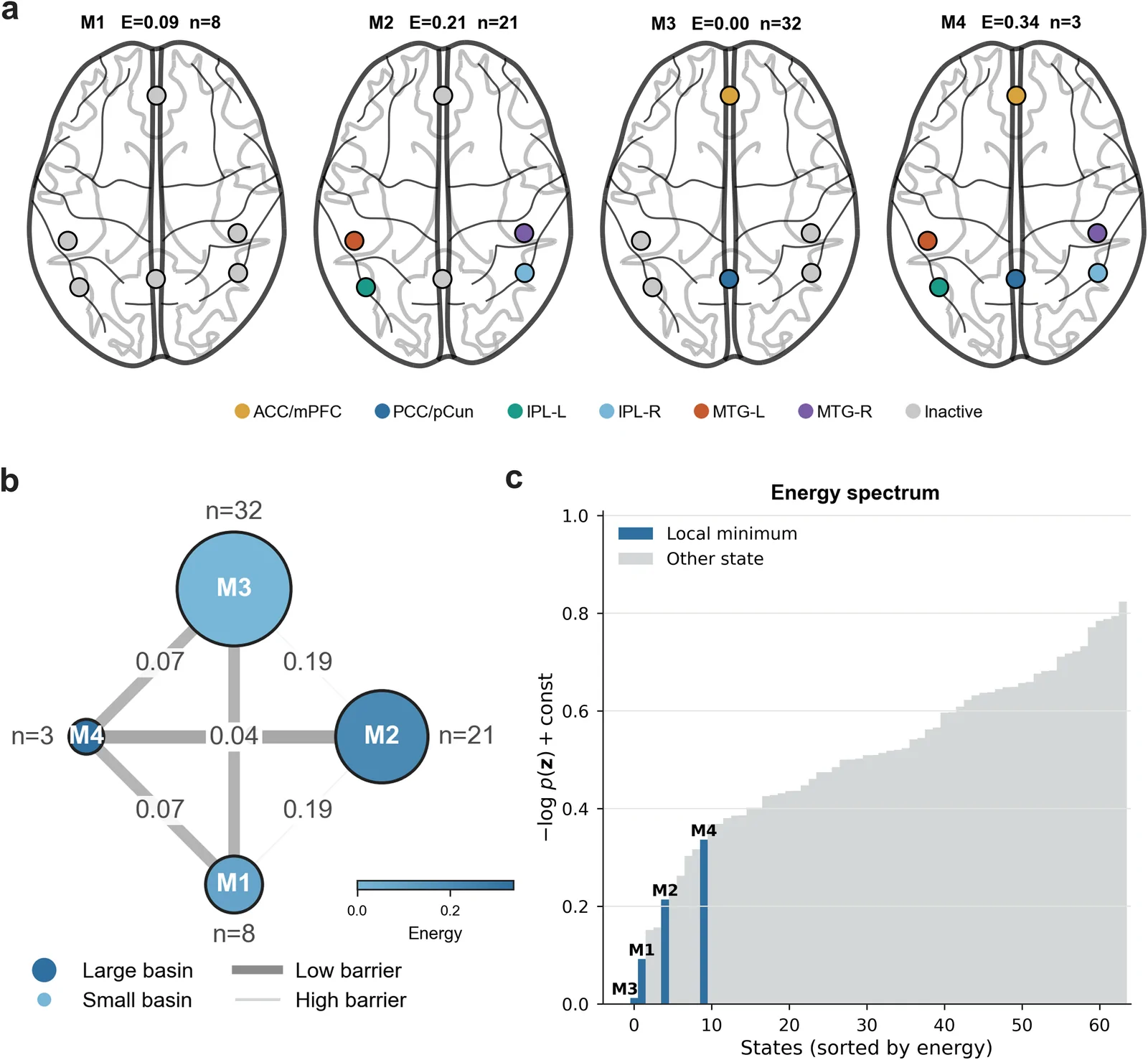
Illustrative pMEM energy landscape for the 6-node DMN computed from a fixed, hand-chosen synthetic (h,J) parameter set, not empirically fitted. Labels M1–M4 identify the four local minima of this specific parameter set and do not represent empirically identified DMN modes. (a) Glass-brain renderings of the four attractor states M1–M4; active nodes are shown in color and inactive nodes in gray. (b) Local-minima network: nodes denote attractor states (size ∝ basin size; color = energy), and edges indicate finite transition barriers. (c) Energy spectrum of all 2^6^ = 64 binary states, with local minima highlighted and labeled.

$$\text{h}={\left(-\;0.149,\;-\;0.065,\;-\;0.294,\;-\;0.408,\;-\;0.171,\;-\;0.336\right)}^{⊤},$$

$$\text{J}=\left(\begin{array}{cccccc} 0 & 0.305 & -0.104 & -0.033 & 0.009 & 0.015\\ & 0 & -0.063 & 0.055 & 0.010 & -0.104\\ & & 0 & 0.347 & 0.015 & 0.237\\ & & & 0 & 0.234 & 0.179\\ & & & & 0 & 0.075\\ & & & & & 0 \end{array}\right),$$

producing a landscape with four local minima, labeled M1–M4; these labels are specific to this illustrative example and do not represent empirically identified DMN modes.

## 3 Results

### 3.1 Simulation of latent states and observed signals

We first verified that the proposed hierarchical pMEM observation model generates continuous signals whose mean structure reflects the underlying latent binary activity. Figure 3 shows an example simulation for an *N* = 8 system with heterogeneous baselines β_0_*_i_* and activation gains β_1_*_i_*. The latent states show stochastic transitions shaped by the pMEM energy landscape, producing region-specific activation bursts.

**Fig. 3.**
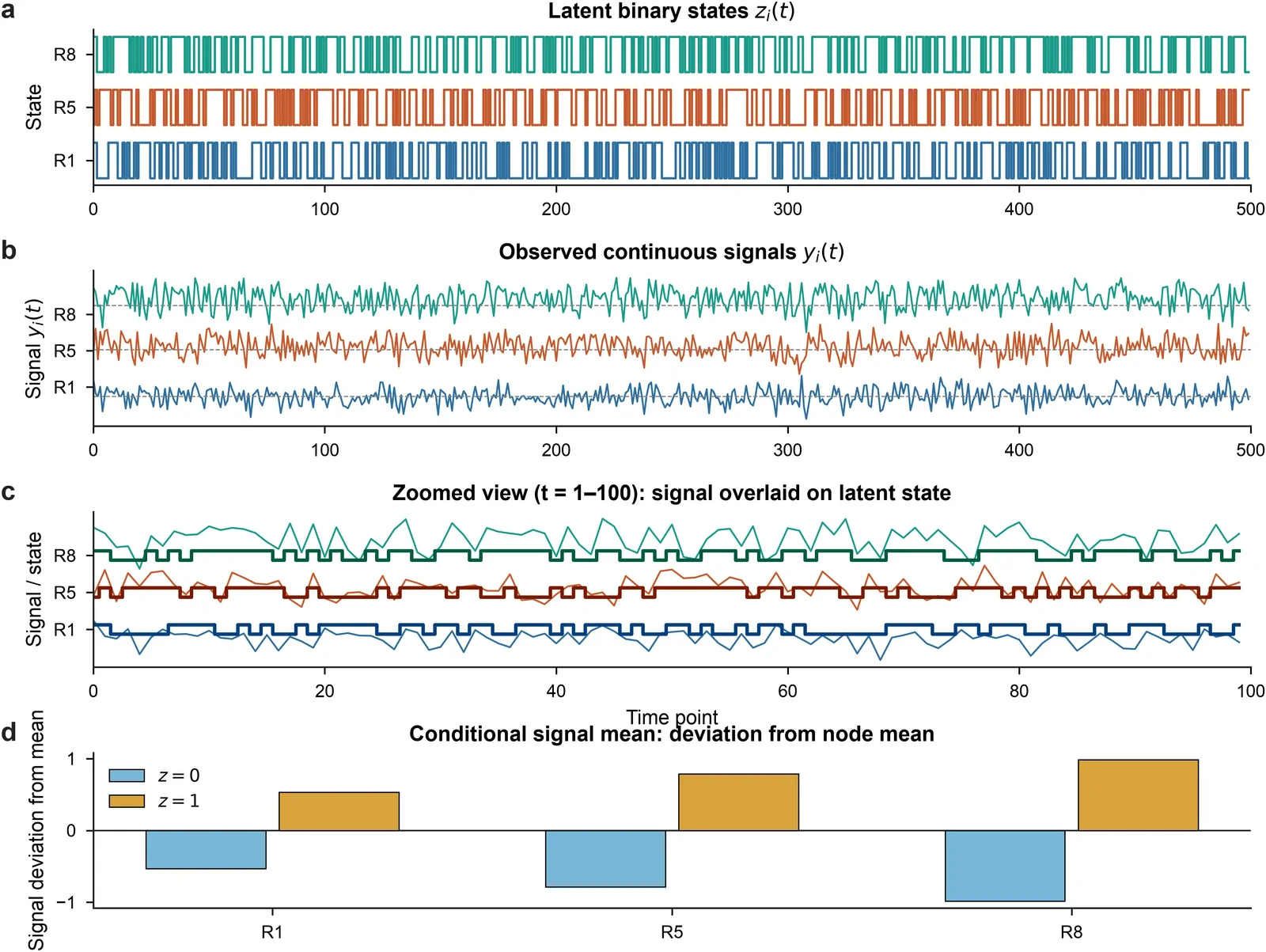
Simulation of latent binary states and observed time series. (a) Latent binary state trajectories *z_i_*(*t*) for three representative nodes (R1, R5, R8). (b) Observed continuous signals $y_{i}\left(t\right)=\beta_{0i}+\beta_{1i}z_{i}\left(t\right)+\varepsilon_{i}\left(t\right)$. (c) Zoomed view (first 100 samples); latent state (step, dark) overlaid on signal (light). (d) Conditional signal means for *z* = 0 (blue) and *z* = 1 (orange), expressed as deviation from the per-node grand mean; positive values indicate the high-amplitude (*z* = 1) state and negative values the low-amplitude (*z* = 0) state.

The observed signals *y_i_*(*t*) followed these latent transitions while retaining continuous amplitude variability. Nodes with larger β_1_*_i_* showed clearer activation-related amplitude shifts, whereas nodes with small or negative baselines remained appropriately centered. A zoomed window illustrates the temporal correspondence between latent states and observations despite multivariate Gaussian noise. Conditioning observations on *z_i_* = 0 versus *z_i_* = 1 further confirmed the expected mean separation, with effect sizes proportional to the true β_1_*_i_*. Thus, the simulation verifies that the observation model produces interpretable continuous time series consistent with the latent pMEM state sequence.

### 3.2 Parameter recovery under cross-channel residual correlation

#### 3.2.1 Recovery evaluation at a representative correlation level

To evaluate whether the proposed pMEM–observation model offers meaningful improvement over the conventional approach that first demeans the data and binarizes the signal at zero, we performed a controlled simulation using known ground-truth parameters. This and the following direct-observation simulations used generic synthetic networks with *N* = 8, unconstrained by any specific anatomical parcellation, to probe parameter recovery independently of the DMN’s particular structure; the 6-node DMN subnetwork (Methods) was instead used for the leadfield simulations and real-data analyses. Latent binary states z(t) were generated from a pMEM with h and J drawn from 10 independently sampled ground-truth instances (*N* = 8; J dense, entries drawn $N\left(0,0.15^{2}\right)$ and symmetrized; h drawn $N\left(0,0.25^{2}\right)$ per node, that is, without a uniform sign bias across nodes, the same multi-instance sampling design used for the residual-correlation sweep and misspecification-robustness checks below (Figs. 5 and 6). Continuous observations were then generated according to

$$\text{y}\left(t\right)=\beta_{0}\;+\beta_{1}⊙\text{z}\left(t\right)+\varepsilon \left(t\right),\;\;\varepsilon \left(t\right)\sim N\left(0,{\text{P}}^{-1}\right),$$

where P has a compound-symmetry structure

$$\text{P}=\frac{1}{\sigma^{2}}\left(\frac{1}{1-\rho }\text{I}-\frac{\rho }{\left(1-\rho \right)\left(1-\rho +N\rho \right)}\text{1}{\text{1}}^{⊤}\right)$$

with noise variance $\sigma^{2}=0.5$ and within-network correlation ρ=0.4 . Both $\beta_{0}$ and $\beta_{1}$ had heterogeneous node-specific values drawn from $N\left(0,\beta_{\text{sd}}^{2}\right)$ added to fixed baselines, with $\beta_{\text{sd}}$ varied across conditions. This creates a setting in which the traditional demeaning and thresholding method is mismatched to the true generative model.

We compared two approaches: (*i*) the conventional demean baseline, that is, demean each node, threshold at zero, pseudolikelihood pMEM; and (*ii*) the proposed β-model, that is, EM using the *exact* posterior over all 2*^ N^* = 256 latent configurations at every E-step (not the mean-field variational approximation described in Methods, which is reserved for networks too large for exact enumeration), jointly estimating $\left(\text{h},\;\text{J},\;\beta_{0},\;\beta_{1}\right)$ with noise parameters estimated from data (no oracle).

We define the “relative error” for a parameter (either the field h or the coupling matrix J) as $∥\hat{\theta }-\theta_{\text{true}}{∥}_{2}/∥\theta_{\text{true}}{∥}_{2}$, where the Euclidean norm is used for h and the Frobenius norm for J. This metric is distinct from the latent-state classification accuracy used to evaluate recovery of z(t).

#### 3.2.2 Recovery evaluation results

Figure 4 shows a representative example of *h* and *J* recovery (Fig. 4a–d) and, aggregating within individual (20 replicates per individual per $\beta_{\text{sd}}$ ) before comparing across the 10 ground-truth instances, summarizes coupling error, joint-distribution divergence, and field recovery (Fig. 4e–g). The demean approach exhibits widespread distortion, particularly in off-diagonal couplings, while the proposed model recovers the dominant interaction structure with markedly lower error.

**Fig. 4.**
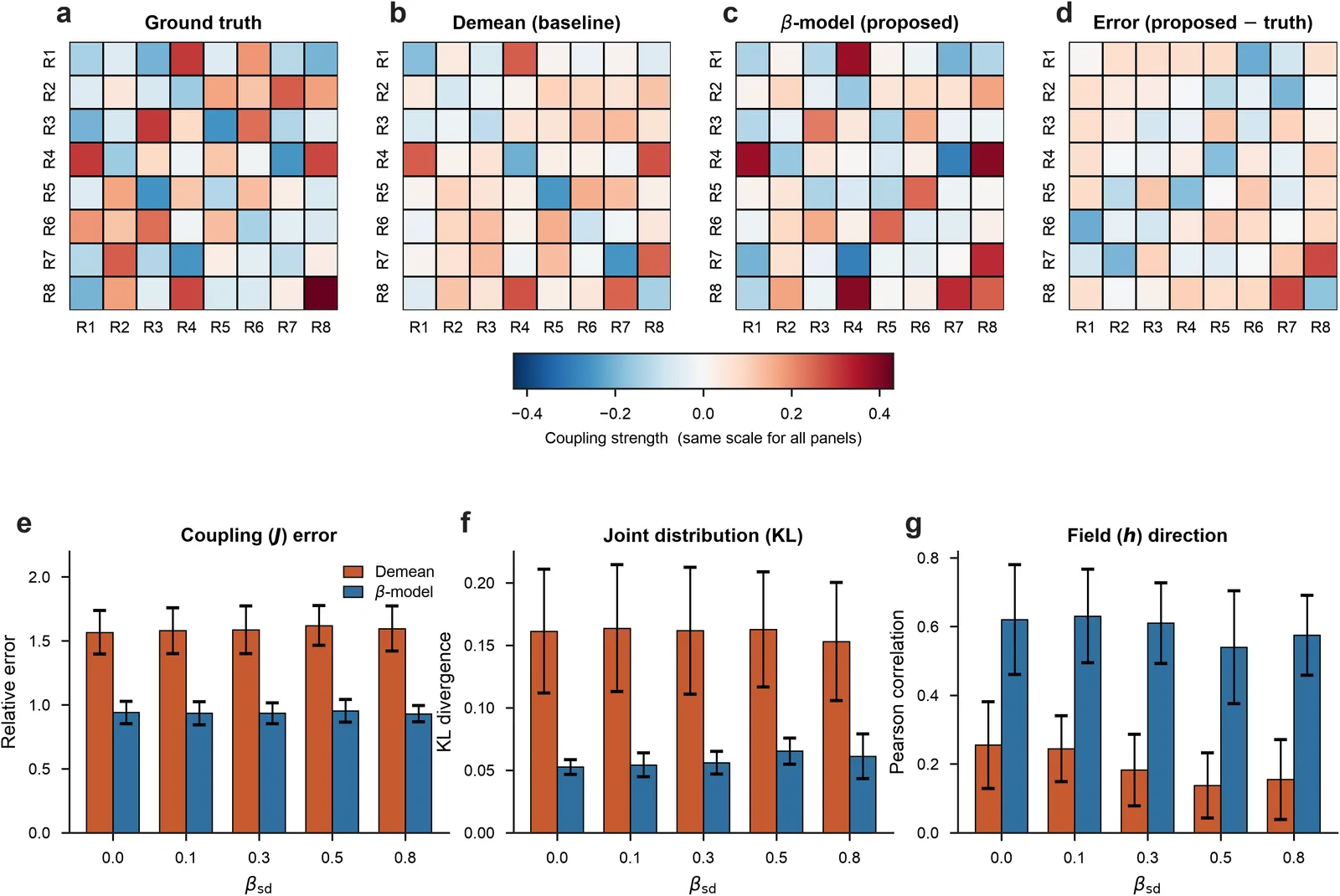
Recovery of pMEM parameters h and J from continuous observations (*N* = 8, *T* = 1000, ρ=0.4 ; 10 independently sampled (h,J) ground-truth instances, 20 replicates per individual per $\beta_{\text{sd}}$ ). (a)–(d) displays a representative case (the ground-truth instance closest to the cross-individual median of $\left(2\times {\text{relerr}}_{J}+{\text{relerr}}_{h}\right)/3$ , which weights J twice h, matching its role as the primary scientific target; not the best-performing instance); (a) Ground truth; (b) Demean baseline (demean → threshold → pseudolikelihood pMEM); (c) Proposed β-model; (d) Pointwise error: proposed − truth. Panels (e)–(g) aggregate within individual (mean over 20 replicates) before summarizing across the 10 ground-truth instances at the main-text $\sigma^{2}\;=0.5$ setting. (e) Coupling (J) relative error across the $\beta_{\text{sd}}$ sweep: proposed model lower at every $\beta_{\text{sd}}$ (e.g. 0.940±0.086 vs. 1.567±0.170 demean at $\beta_{\text{sd}}\;=0$ ; FDR-adjusted p=0.0020 ), underlying the ≈ 41% headline reduction. (f) Exact Kullback–Leibler divergence between the true and fitted pMEM distributions over all 2^8^ = 256 states: favored the proposed model at every $\beta_{\text{sd}}$ (e.g. 0.053±0.006 vs. 0.161±0.050 demean at $\beta_{\text{sd}}\;=0$ , p=0.0020 ; 0.061±0.018 vs. 0.153±0.047 at $\beta_{\text{sd}}=0.8$ , p=0.0020 ). (g) Field (h) Pearson correlation favored the proposed model at every $\beta_{\text{sd}}$ : 0.620±0.160 (proposed) vs. 0.255±0.126 (demean) at $\beta_{\text{sd}}\;=0$ (p=0.0020 ), consistent with h no longer having a near-uniform sign across nodes in this ground-truth design (see above). Bars in (e–g) denote mean ± SD across the 10 individual-level means; all comparisons FDR-corrected across the full 45-test family (5 $\beta_{\text{sd}}$ values × 9 metrics).

Across the 10 ground-truth instances (20 replicates per individual per $\beta_{\text{sd}}$ ; *T* = 1000, ρ=0.4 ; paired Wilcoxon signed-rank tests across the 10 individual-level means), the proposed method reduced field recovery error by ≈ 65% (range 63–66% across $\beta_{\text{sd}}$ ) and coupling error by ≈ 41% (range 40–42%), consistently across all levels of inter-node β heterogeneity ($\beta_{\text{sd}}\in \left\{0.0,\;0.1,\;0.3,\;0.5,\;0.8\right\}$; Fig. 4e). Every one of the nine recovery metrics evaluated (relative error, Pearson correlation, Spearman correlation, and cosine similarity, each for both h and J, plus the exact Kullback–Leibler divergence between the true and fitted pMEM distributions) favored the proposed model at every $\beta_{\text{sd}}$ , all significant after Benjamini–Hochberg false-discovery-rate (FDR) correction across the full 45-test family (5 $\beta_{\text{sd}}$ values × 9 metrics; adjusted p≤0.0039 throughout; Fig. 4f, g). Absolute recovery, nonetheless, remained limited for both methods in this regime: the proposed model’s relative error stayed in the range 0.82–0.95 (only moderately below the trivial zero-parameter baseline of 1.0), while the demean baseline’s relative error exceeded 1.0 throughout, that is, farther from the truth than predicting no field or coupling at all; this reflects the intrinsically weak parameter identifiability of this *N* = 8, *T* = 1000 system rather than a shortcoming specific to either method.

These results establish parameter recovery at the representative correlation level ρ=0.4 . We next examine whether this advantage persists across different levels of residual cross-channel correlation.

#### 3.2.3 Dependence of recovery on residual correlation

We repeated the simulation across ρ∈{0.0, 0.2, 0.4, 0.6, 0.8} (*N* = 8, *T* = 1000, $\sigma^{2}=0.5$ ; single-channel SNR ≈ 2), using 10 independently sampled (h,J) ground-truth instances (100 replicates per individual, 5000 fits per method in total; mean ± SD across the 10 individual-level means, tested by paired Wilcoxon signed-rank tests), to test whether the advantage established above for ρ=0.4 held across the residual-correlation regime (Fig. 5).

**Fig. 5.**
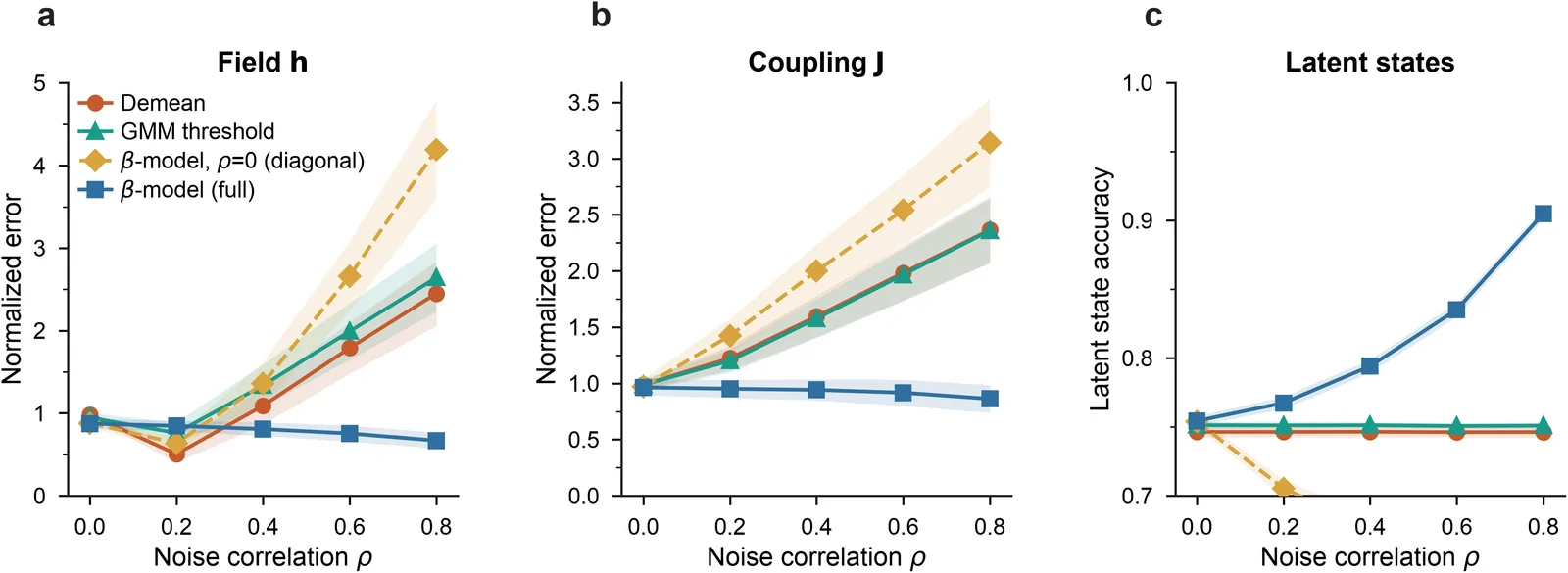
Effect of cross-channel noise correlation ρ on parameter recovery (*N* = 8, *T* = 1000, $\sigma^{2}\;=0.5$ ; 10 independently sampled (h,J) ground-truth instances, 100 replicates per individual). (a) Relative error of the field parameters h: 0.87±0.06 (β-model) vs. 0.98±0.02 (demean) at ρ = 0 (p=0.0020 ); reversed at ρ=0.2 (0.85±0.06 vs. 0.50±0.10 ; p=0.0020 ); β-model favored again beyond ρ≈0.3 (p=0.0098 at 0.4; p=0.0020 at 0.6, 0.8 ), reaching 0.67±0.10 vs. 2.45±0.39 (73% lower) at ρ=0.8 . A control sweep of β-heterogeneity at fixed ρ = 0 showed no comparable reversal, indicating the ρ=0.2 dip reflects weak noise-model identifiability rather than the number of amplitude parameters. (b) Relative error of the coupling parameters J: no reversal at ρ=0.2 ; the β-model was numerically favored throughout, reaching significance for ρ≥0.2 (p≤0.0020 ) but not at ρ = 0 (p=0.1934 ); 0.86±0.12 vs. 2.36±0.30 (64% lower) at ρ=0.8 . (c) Latent-state accuracy: β-model rose from 0.754 to 0.905 across ρ; demean remained flat (≈0.746 ). Between-instance SD was ≤0.009 throughout (c), versus 0.10–0.39 for the field/coupling errors at ρ=0.8 (a,b). Four methods are compared throughout: demean thresholding, per-node GMM thresholding, a diagonal β-model with ρ = 0 fixed, and the full β-model with joint ρ estimation. GMM thresholding performed no better than demeaning and often worse. The diagonal β-model performed worse than demeaning for ρ≥0.2 , reaching 4.19±0.59 vs. 2.45±0.39 field error at ρ=0.8 : amplitude estimation without residual-correlation modeling can amplify rather than reduce noise-induced bias. This pattern, including the transient dip at ρ≈0.2 , was consistent across all 10 independently sampled ground-truth instances.

Field recovery followed a non-monotonic pattern: the full β-model outperformed demeaning at ρ = 0, was transiently outperformed by demeaning at ρ=0.2 (where the off-diagonal residual signal was too weak to offset the cost of jointly estimating the noise parameters, as confirmed by a separate control varying β-heterogeneity at fixed ρ = 0), and then regained an increasing advantage beyond ρ≈0.3 , reaching significance at ρ=0.4 (p=0.0098 ). Coupling recovery showed no such reversal, favoring the full model at every ρ tested. Both advantages were largest at ρ=0.8 (73% and 64% error reduction for field and coupling, respectively). Latent-state classification accuracy was comparatively insensitive to the specific ground-truth instance even where continuous-parameter recovery was not.

A control comparison and an ablation together isolated the source of this advantage. A per-node Gaussian-mixture threshold, which adapts to node-specific offsets and gains but not to shared noise, performed no better than demeaning and often worse. A diagonal β-model, which estimates amplitude parameters but fixes ρ = 0, performed worse than demeaning once ρ≥0.2 and substantially worse at high ρ. Only the joint estimation of amplitude and residual-correlation parameters (neither alone) prevented correlated observation noise from being absorbed into spurious fields and couplings, consistently across all 10 ground-truth instances.

In the real-data analyses (Section 3.6), the estimated $\hat{\rho }$ was well above the ρ≈0.2 transient-reversal regime in every condition, placing these datasets in the regime where the full model’s advantage was consistent for both h and J; this comparison is suggestive only, since the true empirical residual structure and pMEM parameters are unknown.

### 3.3 Robustness and covariance-parameterization checks

The preceding simulations were generated under the assumed model: binary pMEM latent states, compound-symmetry residual covariance, and i.i.d. Gaussian noise. We therefore evaluated two genuine departures from these assumptions (a temporal-autocorrelation violation and a graded-activity violation), together with a complementary consistency check within the compound-symmetry family, an alternative uniform-loading spike parameterization of the residual covariance, each sweeping one parameter while holding all others fixed (*N* = 8, *T* = 1000). Because the primary scientific target is the recovery of inter-node coupling, robustness was assessed primarily using error in J. For all three checks, we first used *n* = 20 replicates from a single fixed (h,J) instance, identical to that used in Figure 5. Because this single-instance design gave an unstable result for the covariance-reparameterization check (see below), we additionally confirmed all three checks using 10 independently sampled (h,J) instances (100 replicates per individual, aggregated within individual and compared across individuals by Wilcoxon signed-rank); for the covariance-reparameterization check, only this multi-instance result is reported below.

#### 3.3.1 Covariance reparameterization check

The true covariance was set to $\Sigma =\sigma^{2}\text{I}+\gamma \text{u}{\text{u}}^{⊤}$ with a uniform loading direction $\text{u}=\text{1}/\sqrt{N}$, so that $\text{u}{\text{u}}^{⊤}\;=\text{1}{\text{1}}^{⊤}\;/N$ and Σ is itself compound-symmetric (equivalent to ρ∈[0, 0.29] for $\sigma^{2}\;=0.5$ , *N* = 8 as γ ranges from 0 to 1.6). This is therefore not a departure from the assumed covariance family, but an alternative, independently-instantiated consistency check of parameter recovery within it, complementary to the ρ-sweep in Figure 5. The β-model was never significantly worse than demeaning at any γ, and was significantly *better* at the largest spike strength tested (γ=1.6 : 28% lower coupling error, FDR-adjusted p=0.0117 ), with the two methods statistically indistinguishable at γ = 0 (Fig. 6a, b). A nominal advantage at γ=0.8 (13% lower error, raw p=0.0195 ) did not survive FDR correction (adjusted p=0.0586 ).

**Fig. 6.**
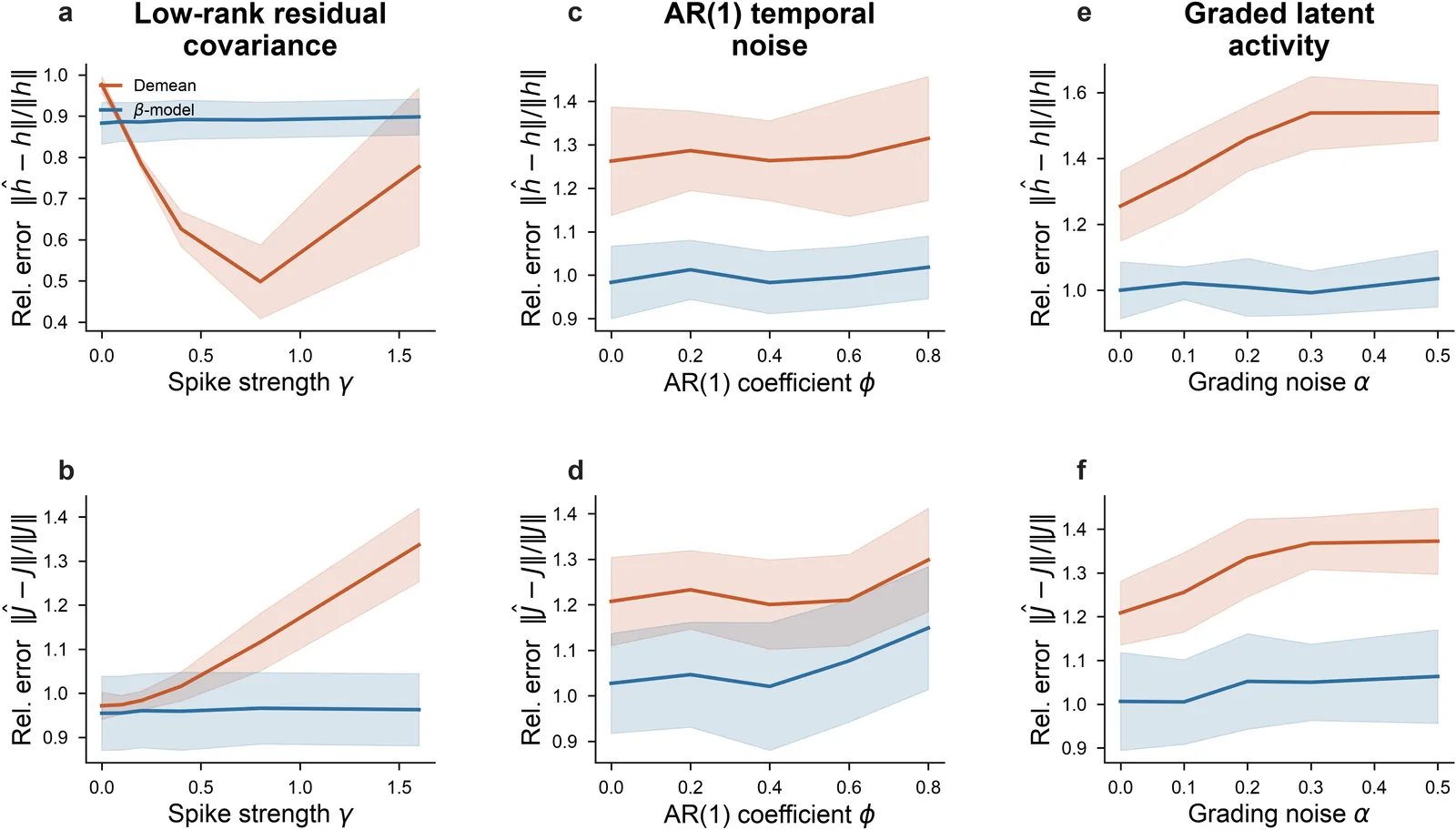
Robustness and covariance-parameterization checks (*N* = 8, *T* = 1000). Each column shows relative recovery error for field parameters h (top) and coupling parameters J (bottom) as one condition is varied. (a, b) Covariance reparameterization check: true $\Sigma =\sigma^{2}\text{I}+\gamma \;\text{u}{\text{u}}^{⊤}$, itself compound-symmetric for the uniform loading u used here; panels show results aggregated across 10 independently drawn (h,J) instances (100 replicates each; mean ± SD across the 10 per-individual means). (c, d) Temporal-autocorrelation violation, AR(1) coefficient ϕ (single (h,J) instance, *n* = 20 replicates). The β-model maintains an 11–15% advantage over demeaning across all ϕ values; temporal autocorrelation does not erode this advantage. (e, f) Graded-activity violation, grading noise α (single (h,J) instance, *n* = 20 replicates). The β-model gains a relative advantage as activity becomes more continuous, because soft posterior inference tracks graded states better than hard thresholding. Shaded bands denote ± 1 SD across replicates (c–f) or across individuals (a, b).

#### 3.3.2 Temporal-autocorrelation violation

The residual noise was drawn from an autoregressive(AR(1)) process $\varepsilon \left(t\right)=\phi \;\varepsilon \left(t-1\right)+\sqrt{1-\phi^{2}}\text{w}\left(t\right)$, w(t)~ $N\left(0,\Sigma_{\text{cs}}\right)$, with compound-symmetry baseline ρ=0.4 and ϕ swept from 0 to 0.8. The recovery advantage was maintained across all tested ϕ, with coupling error reduced by 11–15% relative to demeaning (FDR-adjusted p≤0.002 throughout; Fig. 6c, d).

#### 3.3.3 Graded-activity violation

Latent activity was made continuous by adding Gaussian noise to each binary state, $a_{i}\left(t\right)=\text{clip}\left(z_{i}\left(t\right)+\alpha \;\xi_{i}\left(t\right),0,1\right)$, with $\xi_{i}\left(t\right)\sim N\left(0,1\right)$ and α swept from 0 to 0.5. The β-model’s advantage grew with α, reaching approximately 23% lower coupling error at α=0.5 (FDR-adjusted p<0.001 ; Fig. 6e, f). This pattern is consistent with the soft posterior being less sensitive than hard thresholding to departures from strictly binary activity.

The 10 paired tests for the temporal-autocorrelation and graded-activity violations (five AR(1) coefficients, five graded-activity levels) were corrected for multiple comparisons using the FDR procedure across this family; every significance conclusion reported above was unchanged after correction. Both violations were also confirmed under the 10-individual multi-instance design used for the covariance-reparameterization check: the coupling-error advantage remained significant at every ϕ tested for the temporal-autocorrelation violation (26–35% lower error, p≤0.0039 ) and at every α tested for the graded-activity violation (35–38% lower error, p=0.0020 ), closely reproducing the single-instance pattern reported above for both.

Overall, the proposed model retained its recovery advantage under the temporal-autocorrelation and graded-activity violations (the two genuine model violations tested), and showed no disadvantage, with some benefit at higher spike strength, under the alternative compound-symmetric parameterization examined above.

### 3.4 Lead-field simulation across EEG and MEG modalities

We next evaluated the proposed framework in realistic sensor-space settings using simulated EEG and MEG observations of the form

$$\text{y}\left(t\right)=\text{L}\left(\beta_{0}\;+\beta_{1}⊙\text{z}\left(t\right)\right)+\varepsilon \left(t\right),\;\varepsilon \left(t\right)\sim N\left(0,\Sigma_{\varepsilon }\right),$$

with leadfield matrices derived from the datasets described in Methods (6-node Schaefer 2018 DMN; Section 2.6).

#### 3.4.1 Case 1: 64-channel EEG ($\text{L}\in {\mathbb{R}}^{64\times 6}$ , BioSemi-64, fsaverage three-shell BEM, ρ=0.5 , $\sigma^{2}\text{=0.5}$ )

This setting represents a plausible high-correlation EEG regime under substantial volume conduction (Nunez et al., 1997; Srinivasan et al., 2007). Across 50 replicates (*T* = 1000), the β-model achieved 43% lower field error (1.030 vs. 1.803), 67% lower coupling error (1.189 vs. 3.626), and +12.3 percentage-point higher latent-state accuracy (0.786 vs. 0.663) than the demean+MNE-inverse baseline.

#### 3.4.2 Case 2: MEG magnetometers ($\text{L}\in {\mathbb{R}}^{102\times 6}$ , MNE sample dataset, ρ=0.2 , $\sigma^{2}=0.3$ )

Although ρ=0.2 lies in the weak-correlation regime where the direct-observation analysis showed no net noise-model benefit (Fig. 5), the β-model still improved over the demean+inverse pipeline, with 39% lower field error (0.918 vs. 1.507), 56% lower coupling error (1.252 vs. 2.834), and +8.1 percentage-point higher latent-state accuracy (0.808 vs. 0.727). Because the direct-observation comparison at ρ=0.2 favored demeaning, this MEG gain cannot be attributed to the noise model alone. Instead, it reflects a forward-model advantage: the proposed framework uses L as a generative operator and avoids the source-superposition error introduced by inverse-then-threshold pipelines.

To bound this forward-model advantage, we also included an *oracle source baseline*, in which the true latent source signals

$${\text{S}}_{\text{true}}\left(t\right)=\beta_{0}\;+\beta_{1}⊙\text{z}\left(t\right)$$

were directly demeaned and thresholded before fitting the same pseudolikelihood pMEM. Oracle latent-state accuracy was 1.000 in all replicates, and oracle field errors were 0.620 (EEG) and 0.608 (MEG). Relative to the demean baseline, this defines the full forward-model gap. The proposed β-model closed approximately 65% of this gap in both modalities (65.3% EEG, 65.5% MEG), with the remaining gap reflecting joint uncertainty in $\left(\beta_{0},\;\beta_{1}\right)$ and latent-state inference; unlike the field- and coupling-error reductions above, which differed somewhat between modalities, gap closure relative to the oracle was essentially uniform across EEG and MEG.

These results demonstrate two separable advantages of the proposed framework over the demean-then-invert pipeline: a *noise-model advantage*, evident at sufficiently strong residual correlation, and a *forward-model advantage*, obtained by treating L as a generative forward operator rather than inverting it before binarization. Results are summarized in Figure 7.

**Fig. 7.**
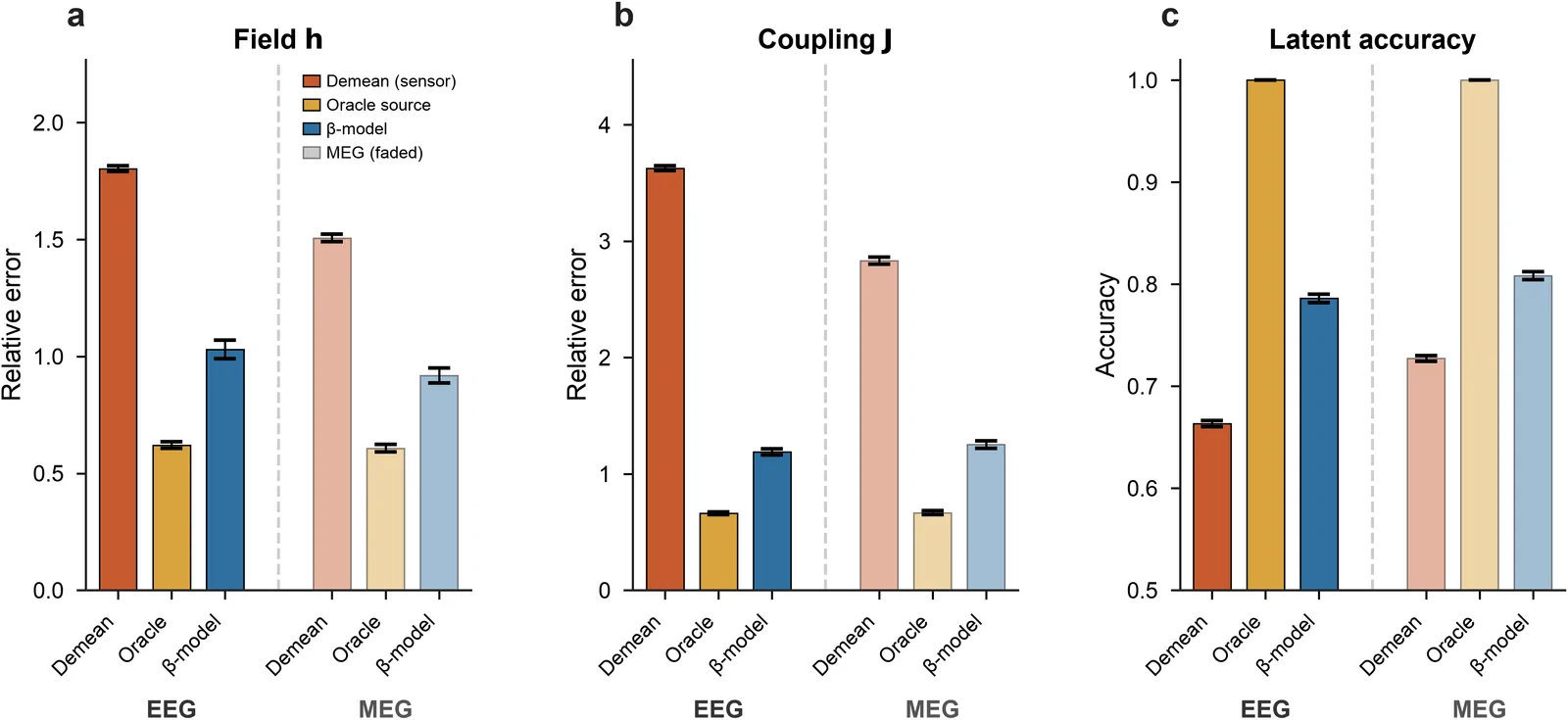
Leadfield pMEM simulation across EEG and MEG modalities (*N* = 6 DMN ROIs, *T* = 1000, *n* = 50 replicates). (a) Field h error. (b) Coupling J error. (c) Latent-state accuracy. Solid bars denote 64-channel EEG (ρ=0.5 ); faded bars denote MEG magnetometers (ρ=0.2 ). Three methods are compared: demean+MNE inverse, oracle source baseline, and the proposed β-model. The oracle defines the ceiling of the forward-model advantage, and the proposed model closes a substantial fraction of the demean-to-oracle gap in both modalities. Error bars indicate ±SEM.

### 3.5 Resting-state EEG validation: PhysioNet dataset

We applied the β-model to the PhysioNet EEG dataset (Methods; *N* = 20 subjects, eyes-open and eyes-closed baseline runs) as a real-data stability and interpretability check. Because ground-truth parameters are unavailable, the aim was not parameter recovery but to assess whether the model returns plausible residual-correlation estimates and interpretable energy-landscape summaries.

The estimated residual correlation was high in both conditions, with $\hat{\rho }=0.841\pm 0.127\;\text{(eyes-open)}$ and $\hat{\rho }=0.895\pm$ 0.027 (eyes-closed), with a significant difference between conditions (Wilcoxon p<0.001 ). Both values lie well above the weak-correlation regime identified in simulation. Here, however, $\hat{\rho }$ represents effective residual spatial correlation after the 6-node DMN signal has been modeled, likely reflecting unmodeled non-DMN alpha activity.

The number of local minima showed a non-significant trend toward more fragmented landscapes during eyes-closed rest (10.7±5.5 eyes-open vs. 11.7±2.2 eyes-closed; Wilcoxon p=0.057 ), and basin entropy $H_{\text{basin}}$ did not differ significantly between conditions (1.399±0.347 vs. 1.540±0.143 ; Wilcoxon p=0.123 ; Fig. 8b). The source-wise mean field parameters h showed broadly similar spatial profiles across the two conditions, without a consistent condition-wide shift (Fig. 8c). The proposed model can therefore be fitted stably to empirical EEG and returns plausible residual-correlation estimates across conditions, with landscape summaries that trend but do not reach significance between conditions in this dataset.

**Fig. 8.**
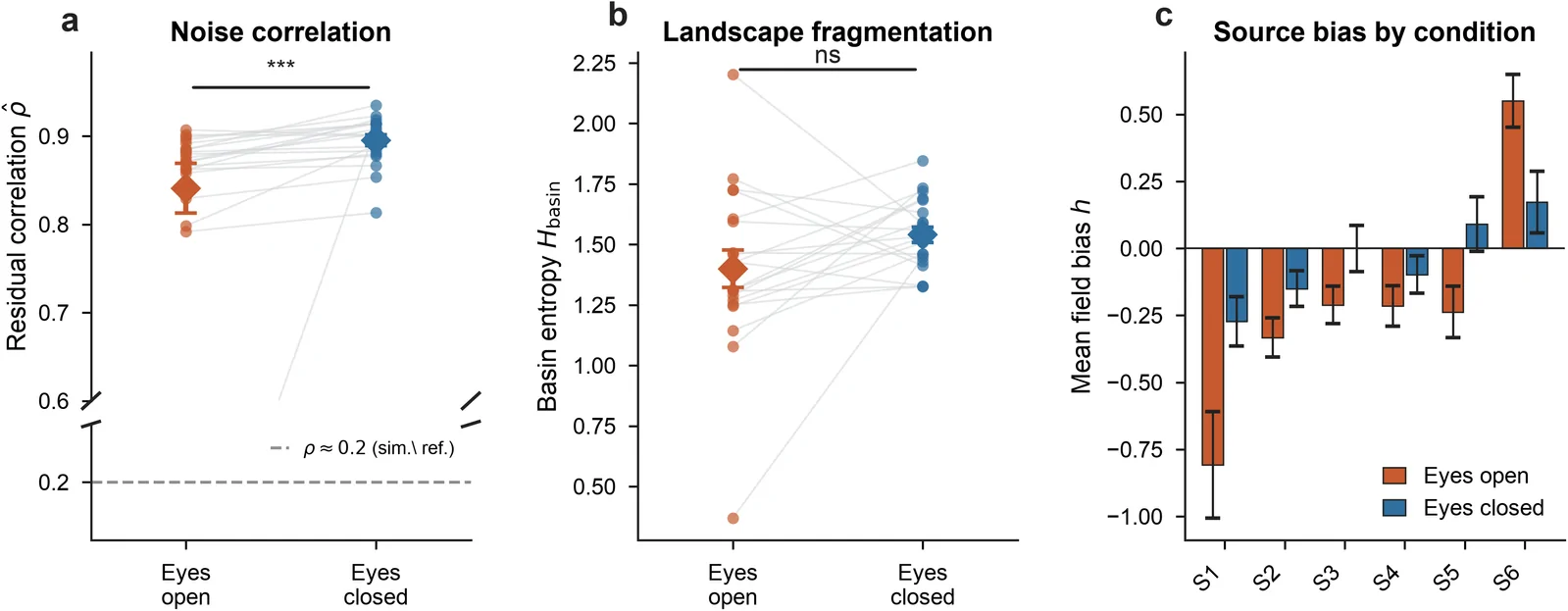
PhysioNet resting-state EEG validation. (a) Estimated residual correlation $\hat{\rho }$ for eyes-open and eyes-closed rest; eyes-closed significantly higher (Wilcoxon p<0.001 ). Both conditions lie well above the weak-correlation regime identified in simulation; the high values likely reflect unmodeled non-DMN alpha activity. (b) Basin entropy $H_{\text{basin}}$ per subject for eyes-open and eyes-closed rest; no significant difference between conditions (Wilcoxon p=0.123 ). (c) Mean field bias h for each DMN source under the two conditions. ***p < 0.001.

### 3.6 DMN energy landscape changes under propofol sedation

#### 3.6.1 Motivation and dataset

A primary motivation for fitting the pMEM energy landscape is to detect condition-dependent changes in attractor structure that are not readily captured by standard spectral or connectivity measures. Propofol-induced sedation provides a controlled test case because the drug suppresses consciousness in a dose-dependent manner (Alkire et al., 2008), and theories of anesthetic action predict altered stability and accessibility of large-scale cortical states rather than uniform suppression (Mashour et al., 2020; Tononi, 2008).

We applied the β-model to the Chennu propofol sedation EEG dataset (Methods; *N* = 20 healthy volunteers, four conditions: baseline, mild sedation, moderate sedation, and recovery; 91-channel Electrical Geodesics Inc. (EGI) system, alpha-band amplitude envelopes 8–13 Hz). Across all three summary measures examined below (residual correlation, basin entropy, and the number of local minima), the proposed model detected significant condition-dependent effects (all p≤0.03 ), whereas the conventional demean baseline detected none (all p≥0.15 ); as detailed below, this indicates the inferred landscape depends materially on the observation-to-latent-state pipeline, without by itself establishing which pipeline is closer to the unknown biological ground truth.

#### 3.6.2 Residual correlation across sedation levels

The estimated residual correlation $\hat{\rho }$ showed a significant condition-dependent inverted-U pattern across baseline, mild sedation, moderate sedation, and recovery: 0.789±0.020,  0.797±0.024,  0.808±0.025,  0.790±0.025, respectively (Friedman $\chi^{2}\;=16.14$ , p=0.0011 ). The ascending phase from baseline to moderate sedation was supported by Page’s trend test (p<0.0001 ), and the return from moderate sedation to recovery was significant (Wilcoxon p=0.0020 ), indicating that the fitted residual-correlation parameter returned toward its baseline level during recovery. All conditions lay well above the ρ≈0.2 weak-correlation regime identified in simulation. However, $\hat{\rho }$ does not represent pure sensor noise or volume conduction; rather, it represents effective residual spatial correlation after the 6-node DMN source activity has been modeled, likely including correlated non-DMN alpha activity absorbed into the residual term (Chennu et al., 2016; Cimenser et al., 2011; Purdon et al., 2013).

#### 3.6.3 Energy-landscape fragmentation

Basin entropy $H_{\text{basin}}$ and the number of local minima *n_min_* (both defined in Section 2.7) quantify complementary aspects of landscape structure. Both showed a significant condition-dependent effect across sedation levels (*n_min_*: Friedman $\chi^{2}\;=9.07$ , p=0.0284 ; $H_{\text{basin}}$ : Friedman $\chi^{2}\;=8.82$ , p=0.0318 ; Fig. 9). For *n_min_*, values followed an inverted-U pattern similar to $\hat{\rho }$ : 17.5±3.3 , 19.2±4.5 , 21.1±3.7 , and 18.1±3.7 at baseline, mild, moderate, and recovery, respectively, with a significant rise from baseline to moderate sedation (p=0.0114 ) and a significant fall from moderate sedation to recovery (p=0.0251 ); baseline-to-mild and baseline-to-recovery differences were not significant (p=0.1757 and p=0.3639 ). $H_{\text{basin}}$ , by contrast, did not follow a simple inverted-U shape: values were 1.288±0.379 at baseline, 1.534±0.254 at mild sedation, 1.278±0.391 at moderate sedation, and 1.501±0.230 at recovery. Entropy at recovery was significantly elevated relative to both baseline (p=0.0064 ) and moderate sedation (p=0.0240 ), while baseline and moderate sedation did not differ from each other (p=1.0000 ). Mild sedation was nominally elevated relative to baseline (p=0.0484 ), but this comparison was not among the tests included in the FDR correction family and was not directly tested against moderate sedation (see below); we therefore treat it as an exploratory observation rather than a confirmed effect. We do not have a mechanistic account of why landscape fragmentation would be elevated at recovery but not at moderate sedation, and treat this pattern as hypothesis-generating rather than a confirmed dose-response relationship.

**Fig. 9.**
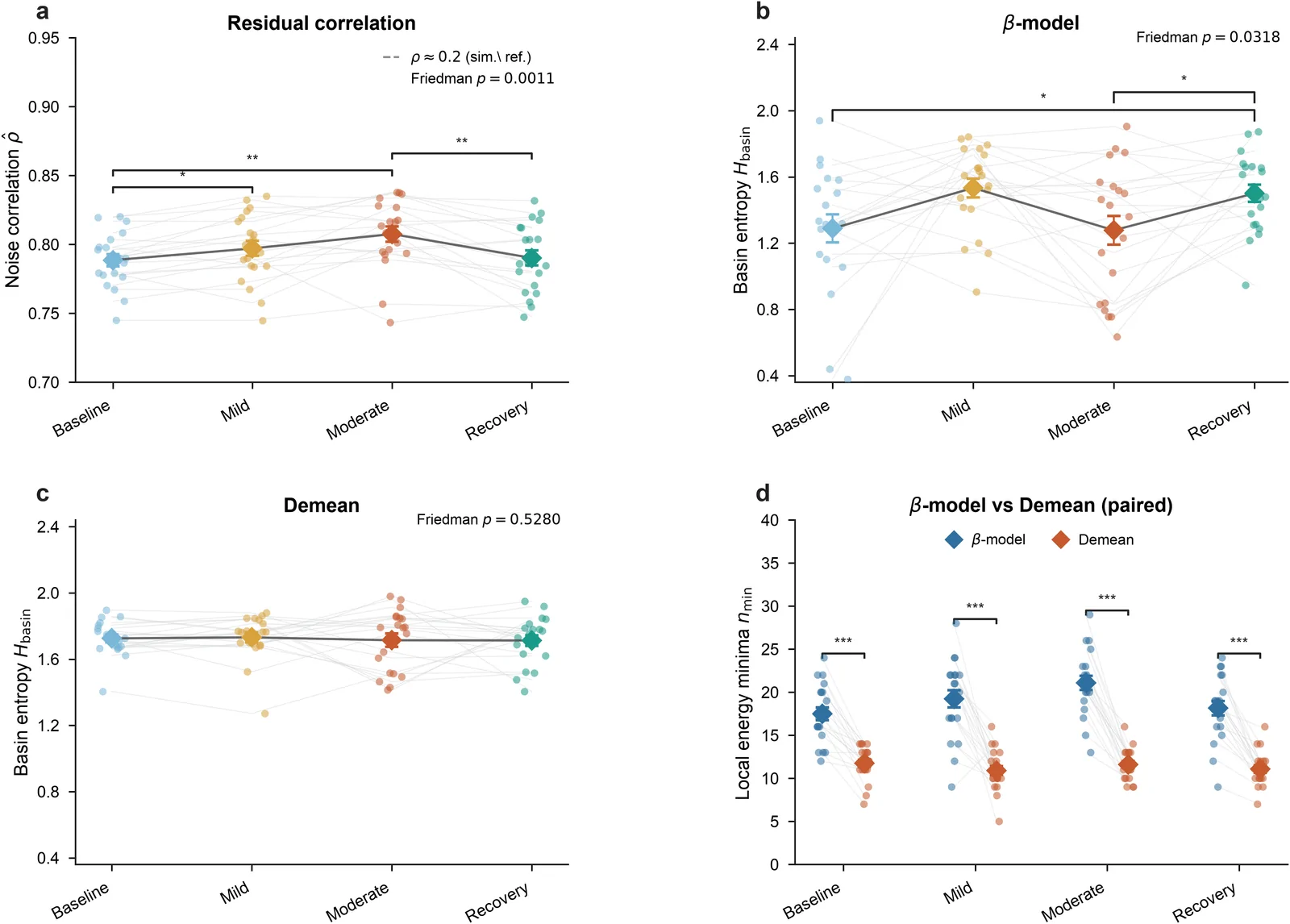
DMN energy landscape changes under propofol sedation (*N* = 20, Chennu dataset (Chennu et al., 2016)). (a) Estimated residual correlation $\hat{\rho }$ across sedation conditions, showing an inverted-U profile peaking at moderate sedation and returning during recovery. (b) Basin entropy $H_{\text{basin}}$ per subject for the β-model. Landscape fragmentation showed a significant non-monotonic pattern (Friedman p=0.0318 ), with entropy at recovery significantly elevated relative to baseline (p=0.0064 ) and moderate sedation (p=0.0240 ); mild sedation was nominally elevated relative to baseline (p=0.0484 , uncorrected). (c) Basin entropy $H_{\text{basin}}$ per subject for the demean pipeline. Values showed little variation across the four conditions (1.713–1.731 ), and the Friedman test was not significant (p=0.528 ). (d) Within-subject comparison of the number of fitted local energy minima between the β-model and demean pipeline. The β-model produced more local minima at each condition (Wilcoxon signed-rank; p≤0.0002 at every condition). *p < 0.5, **p < 0.01, ***p < 0.001.

Nine inferential tests were included in FDR correction family: Friedman on $\hat{\rho }$ , Page trend, and Wilcoxon moderate vs. recovery for $\hat{\rho }$ ; Friedman on *n_min_*, and Wilcoxon baseline vs. moderate and moderate vs. recovery for *n_min_*; and Friedman on basin entropy, and Wilcoxon baseline vs. recovery and moderate vs. recovery for basin entropy. All nine tests survived FDR at q<0.05 ; the largest unadjusted *p*-value in the family was 0.0318 (basin-entropy Friedman), and all nine remained significant after correction. The baseline vs. moderate basin-entropy comparison, not among these nine tests, remained non-significant; the baseline vs. mild basin-entropy comparison was nominally significant before correction (p=0.0484 ) but, being likewise outside this set, is reported as an exploratory, uncorrected result.

#### 3.6.4 Comparison with the demean baseline

A direct comparison with the conventional demean pipeline is shown in Figure 9b–d. The demean pipeline, defined as MNE minimum-norm inverse followed by demeaning, thresholding, and pseudolikelihood pMEM estimation, produced basin entropy values of 1.713–1.731 across all four sedation conditions, showing no significant change (Friedman $\chi^{2}\;=2.22$ , p=0.528 ; Fig. 9c). The demean pipeline’s number of local minima likewise showed no significant condition effect (11.8±1.9 , 10.9±2.4 , 11.6±1.8 , 11.1±1.9 at baseline, mild, moderate, and recovery; Friedman $\chi^{2}\;=5.32$ , p=0.1496 ). In contrast, the β-model basin entropy varied from 1.278 at moderate sedation to 1.534 at mild sedation, yielding a significant Friedman effect (p=0.0318 ; Fig. 9b), and its number of local minima also showed a significant condition effect (p=0.0284 , reported above). Because the same subjects, node definition, conditions, and Boltzmann-weighted basin summary were used for both pipelines, this comparison shows that the inferred landscape result depends materially on the observation-to-latent-state inference pipeline. It does not establish which pipeline is closer to the unknown biological ground truth, nor that the β-model is more statistically sensitive in general. The β-model also yielded more fitted local minima than the demean pipeline at every condition (Wilcoxon signed-rank; 17.5±3.3 vs. 11.8±1.9 at baseline, 19.2±4.5 vs. 10.9±2.4 at mild, 21.1±3.7 vs. 11.6±1.8 at moderate, 18.1±3.7 vs. 11.1±1.9 at recovery; p≤0.0002 at each condition). This indicates that the two pipelines produced systematically different static landscape structures; it does not demonstrate richer temporal state-space exploration.

#### 3.6.5 Sensitivity to network definition and frequency band

We repeated the propofol analysis using three alternative configurations: a 6-node Frontoparietal Control Network (FPN-6) in the alpha band, the original DMN-6 in the 30–45 Hz gamma band, and a spatially clustered 6-node visual network (Vis-6) in the alpha band, each using the same 20 subjects, four conditions, and processing pipeline as the primary DMN-6/alpha analysis.

The condition effect on residual correlation remained significant in all three configurations, matching the primary DMN-6/alpha result above (FPN-6: $\hat{\rho }=0.799/0.804/$ 0.815/0.798 , Friedman $\chi^{2}\;=13.62$ , p=0.0035 ; DMN-6/gamma: $\hat{\rho }=0.733/0.746/0.769/0.726$ , $\chi^{2}\;=14.58$ , p=0.0022 ; Vis-6: $\hat{\rho }=0.784/0.788/0.804/0.776$ , $\chi^{2}\;=16.50$ , p=0.0009 ), with $\hat{\rho }$ increasing from baseline to moderate sedation and decreasing during recovery in every configuration. The clustered Vis-6 network had the highest mean pairwise leadfield-column correlation of the four node sets (0.48 vs. 0.29–0.31) but did not show a distinctly stronger $\hat{\rho }$ effect.

In contrast, the condition effect on basin entropy did not reach significance in any of the three configurations (FPN-6: $\chi^{2}\;=5.22$ , p=0.1564 ; DMN-6/gamma: $\chi^{2}\;=2.94$ , p=0.4010 ; Vis-6: $\chi^{2}\;=0.54$ , p=0.9100 ), unlike the primary DMN-6/alpha analysis ($\chi^{2}\;=8.82$ , p=0.0318 ). The residual-correlation effect was therefore not specific to the primary DMN-6/alpha configuration, whereas the basin-entropy effect was. Because these are empirical analyses without ground truth, they assess the consistency of the condition effects rather than estimation accuracy. None of the tests in this paragraph belong to the nine-test FDR family defined above for the primary DMN-6/alpha analysis; all *p*-values here are exploratory and uncorrected for multiple comparisons.

## 4 Discussion

This study reframes conventional pMEM binarization as a restrictive hard-assignment limit of a broader generative observation model for continuous neuroimaging data. In the standard demean-and-threshold pipeline, the baseline is fixed to the empirical channel mean, amplitude gain and residual covariance are not explicitly modeled, binary states are assigned deterministically, and the pMEM energy landscape is estimated only after this preprocessing step. In the proposed framework, binary network states are treated as latent variables and inferred jointly with baseline and gain parameters, residual precision, and pMEM parameters. Thus, binarization becomes an inference problem embedded within the pMEM model itself.

This formulation yielded two separable advantages. The first was a noise-model advantage: when residual fluctuations were spatially shared, conventional thresholding could convert observation-level covariance into apparent multi-node co-activation, distorting fields, couplings, and attractor structure. By modeling residual precision explicitly, the proposed framework could assign shared fluctuations to the observation-noise term rather than to the latent pMEM state. This benefit was regime-dependent, with limited advantage when residual correlation was weak and difficult to identify, but clear improvement when residual dependence was sufficiently strong. The second was a forward-model advantage: for EEG/MEG, treating the leadfield as a generative operator allowed latent binary states to be inferred directly from sensor-space observations, avoiding the source-superposition errors introduced by inverse-first thresholding pipelines.

We present the empirical analyses as proof-of-concept demonstrations rather than definitive neurobiological tests. In the PhysioNet resting-state EEG data, the model produced stable fits and interpretable residual-correlation and landscape summaries. In the propofol sedation dataset, the two observation-to-latent-state pipelines produced different condition-dependent landscape results: the β-model showed significant condition effects in its fitted residual-correlation parameter, basin entropy, and the number of local minima. For the two landscape summaries, the conventional demean-based pipeline did not show significant condition effects. Because the biological ground truth is unavailable, this comparison demonstrates that the inferred landscape depends on the observation-to-latent-state pipeline, rather than establishing comparative accuracy.

The two landscape summaries did not follow the same pattern as each other: the number of local minima followed an inverted-U shape similar to $\hat{\rho }$ , peaking at moderate sedation, whereas basin entropy was instead significantly elevated at recovery relative to both baseline and moderate sedation, with a further nominal, uncorrected elevation at mild sedation relative to baseline, a non-monotonic pattern suggesting that landscape organization does not follow a simple dose-response relationship. The fitted $\hat{\rho }$ represents effective residual spatial dependence after accounting for the modeled sources.

This recovery-condition finding is not a complete characterization of post-anesthetic dynamics, since only one recovery timepoint was analyzed. The PhysioNet resting-state data showed significantly higher residual correlation in eyes-closed than eyes-open rest, consistent with elevated and spatially coherent alpha activity after visual input is removed, whereas basin entropy did not differ significantly between conditions (full statistics in Section 3.5). This interpretation is broadly compatible with theories linking anesthetic loss and recovery of consciousness to altered large-scale integration and cortical dynamics (Alkire et al., 2008; Casali et al., 2013; Mashour et al., 2020; Tagliazucchi et al., 2016; Tononi, 2008), though the present data do not establish a DMN-specific mechanism of anesthetic state transitions.

Several limitations define the scope of the present framework.

First, the benefit of residual-covariance modeling depends on the residual-correlation regime; when residual correlation is weak, estimating covariance parameters can introduce overhead.

Second, the compound-symmetry covariance model is intentionally parsimonious and cannot capture structured residual sources such as non-DMN alpha topographies, reference-dependent EEG covariance, or distance-dependent sensor correlations; more flexible structured covariance bases (e.g., distance-banded components) could capture such effects within the same EM framework, although they would require numerical rather than closed-form precision-matrix updates and additional regularization.

Third, exact inference and exhaustive landscape enumeration both scale exponentially with network size, since both require summation over all 2*^ N^* configurations: exact per-time-point posterior inference costs *O*(*T*·2*^N^*), while exhaustive enumeration of the fitted landscape (identifying local minima and basins) is a one-time cost of *O*(*N*·2*^N^*), independent of *T*. In the present implementation, exact analyses were practical for the small networks used here (*N* = 6–8); a single-run benchmark places the practical ceiling for exact inference at approximately *N* ≈ 14–16 nodes on standard hardware, with cost roughly doubling per additional node (order-of-magnitude estimate only). The mean-field extension improves scalability for parameter inference but does not by itself make exhaustive attractor/basin enumeration tractable; substantially larger networks would require approximate inference combined with targeted landscape-characterization methods. The mean-field extension was derived but not benchmarked against exact enumeration in this study, and its empirical accuracy for larger or strongly coupled networks remains to be established.

Fourth, the observation model assumes temporal independence given the latent states, which simplifies inference but does not capture autocorrelation in EEG envelopes or fMRI signals; the optimal downsampling rate that best satisfies this independence assumption warrants further investigation. Relatedly, basin entropy and the number of local minima describe the static structure of the fitted energy landscape (how probability mass is distributed across attractor basins) but say nothing about the actual dwell times, transition rates, or trajectories through that landscape; landscapes with similar basin structure can therefore support qualitatively different dynamics, and a full dynamical characterization would require complementary trajectory-based analyses (e.g., transition-rate or dwell-time estimation) beyond the static summaries used here.

Fifth, because alpha-band amplitude envelopes are obtained after filtering and Hilbert transformation, the leadfield serves here as an effective forward model for spatially mixed envelope observations. The underlying EEG/MEG measurements are linearly mixed by the electromagnetic leadfield, and the resulting alpha-envelope features inherit this spatial mixing. Thus, incorporating L provides a first-order correction for source superposition in the continuous observations used for pMEM inference.

Sixth, although the continuous parameter estimates were stable across restarts in the tested datasets, discrete landscape summaries were more sensitive to optimization settings. In a restart-sensitivity analysis of five propofol subjects with 10 restarts each, the EM objective varied by less than 0.04% per subject, and the recovered (h,J) showed high agreement with the best-objective solution (r=0.94 –1.00). In contrast, *n_min_* varied by 2–3 across restarts for most subjects. The 600-iteration budget used in the leadfield analyses was selected from convergence checks over 150–1500 iterations and was sufficient for stabilization of the monitored objective and noise parameters; however, discrete landscape summaries may remain more sensitive to the fitting budget. When local-minima counts or basin-based summaries are primary outcomes, we therefore recommend fitting 5–10 restarts with different random J perturbations, retaining the solution with the highest EM objective, and confirming that the statistic of interest is stable across both restarts and longer iteration budgets.

Seventh, the alternative-network and frequency-band analyses (Section 3.6) assess generalizability and practical sensitivity of the proposed model’s empirical findings rather than ground-truth estimation accuracy, which is established only through the simulations above. We examined one spatially distributed network (frontoparietal) and one spatially clustered network (visual), plus a frequency-band check (gamma), as representative cases; extension to other clustered sensory networks such as motor-sensory and auditory is left for future work.

Finally, the primary focus of this study is the estimation of the pMEM parameters that define the energy landscape from continuous observations; the empirical analyses examined only two selected structural features of the fitted landscape, the number of local minima and basin entropy, which do not provide a complete description of temporal dynamics for the reasons given in the fourth point above.

The proposed model is distinct from node-wise soft-thresholding or Gaussian mixture approaches. Per-node mixture models infer each *z_i_*(*t*) marginally from its own observation *y_i_*(*t*), without a joint pMEM prior over the full state vector. In contrast, the proposed framework infers the joint posterior p(z(t)|y(t)) under the pMEM prior while estimating the observation parameters. The interaction matrix J therefore shapes latent-state inference directly, allowing the model to distinguish correlated observation noise from genuine multi-node co-activation.

In summary, conventional demeaning and thresholding can be viewed as a limiting case of a continuous-observation generative model for pMEM estimation. By relaxing this limiting case, the proposed framework jointly estimates latent binary states, observation parameters, residual covariance, and pMEM energy landscapes. The framework links continuous EEG/MEG or fMRI observations to energy-landscape analysis without imposing a fixed threshold, while making explicit when conventional thresholding is likely to be adequate and when its assumptions may break down.

## Data Availability

The PhysioNet EEG Motor Movement/Imagery dataset is freely available at https://physionet.org/content/eegmmidb/1.0.0/ (Goldberger et al., 2000; Schalk, 2009). The MNE sample MEG dataset is distributed with MNE-Python and is available at https://mne.tools/stable/index.html (Gramfort et al., 2014). The propofol sedation EEG dataset (Chennu et al., 2016) is openly available from the University of Cambridge Data Repository at https://doi.org/10.17863/CAM.68959, under a Creative Commons Attribution 2.0 UK: England & Wales license. The simulation and analysis code is available at https://github.com/psalmhim/pMEM-EEGMEG, implemented in Python (≥3.9) using NumPy (≥1.23), SciPy (≥1.9), scikit-learn (≥1.1), MNE-Python (≥1.3), Nilearn (≥0.10), and Matplotlib (≥3.6); exact package versions are pinned in requirements.txt in the repository.
